# Monatomic reactions with single vacancy monolayer h-BN: DFT studies†

**DOI:** 10.1039/d3ra05108k

**Published:** 2023-10-16

**Authors:** Nicholas Mondinos, Mohammednoor Altarawneh, Amun Amri, Willey Yun Hsien Liew, Gerrard Eddy Jai Poinern, Zhong-Tao Jiang

**Affiliations:** a Surface Analysis and Materials Engineering Research Group, School of Mathematics, Statistics, Chemistry and Physics, College of Science, Technology, Engineering and Mathematics, Murdoch University Murdoch WA 6150 Australia Z.Jiang@murdoch.edu.au; b Department of Chemical and Petroleum Engineering, United Arab Emirates University 15551 United Arab Emirates mn.altarawneh@uaeu.ac.ae; c Department of Chemical Engineering, Universitas Riau Pekanbaru Indonesia; d Faculty of Engineering, Universiti Malaysia Sabah Jalan UMS 88400 Kota Kinabalu Sabah Malaysia; e Murdoch Applied Innovation Nanotechnology Research Group, School of Mathematics, Statistics, Chemistry and Physics, College of Science, Technology, Engineering and Mathematics, Murdoch University Murdoch WA 6150 Australia

## Abstract

Hexagonal boron nitride (h-BN) has been widely utilized in various strategic applications. Fine-tuning properties of BN towards the desired application often involves ad-atom adsorption of modifying its geometries through creating surface defects. This work utilizes accurate DFT computations to investigate adsorption of selected 1st and 2nd row elements (H, Li, C, O, Al, Si, P, S) of the periodic table on various structural geometries of BN. The underlying aim is to assess the change in key electronic properties upon the adsorption process. In addition to the pristine BN, B and N vacancies were comprehensively considered and a large array of properties (*i.e.*, atomic charges, adsorption energies, density of states) were computed and contrasted among the eight elements. For instance, we found that the band gap to vary between 0.33 eV (in case of Li) and 4.14 eV (in case of P). Likewise, we have illustrated that magnetic contribution to differ substantially depending on the adatom adsorbents. Results from this work has also lays a theoretical foundation for the use of decorated and defected BN as a chemical sensor for CO gases.

## Abstract 2

Single atom chemisorption of hexagonal boron nitride (h-BN) result with a range of physicochemical properties for various strategic applications.

## Graphical abstract

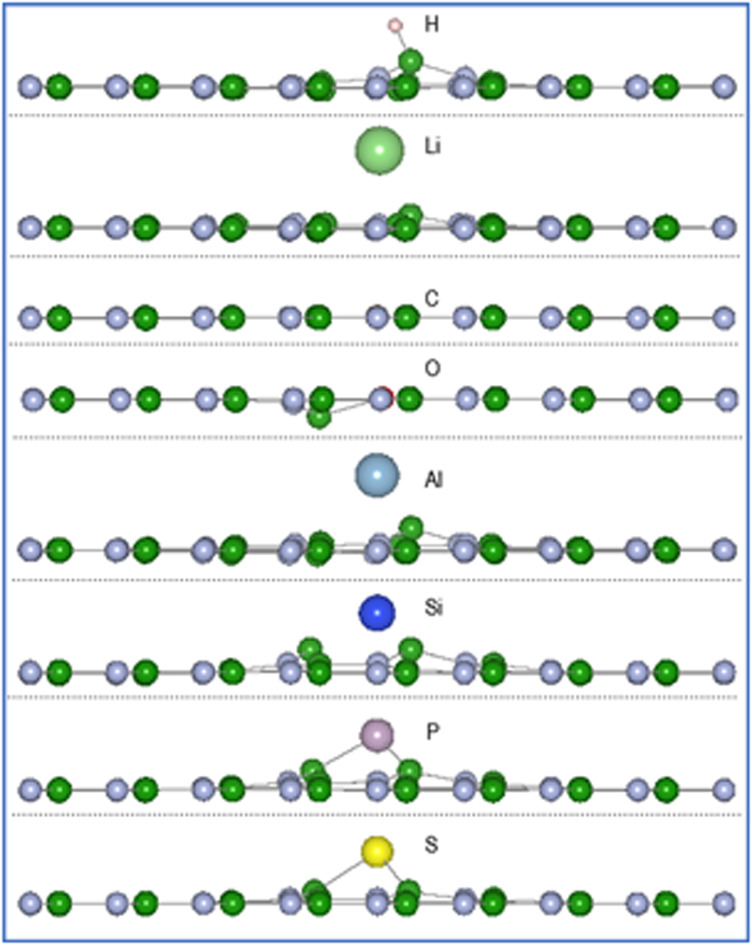

## 1. Introduction

Hexagonal boron nitride (h-BN), isostructural to semi-metallic graphite, and in the form of a two-dimensional (2D) sheet has added complexity in its physical and chemical properties and is similar in its crystal structure and synthetic production with other 2D structures as reviewed by Tan *et al.*^1^ A perspective by Zhang,^2^ gives an overall picture of the usefulness and preparation for the unique group of 2D nanomaterials that h-BN is a valuable member for several promising new applications. Boron nitride (BN) has been fabricated into various form that include single to multi-layer sheets, ribbons, and nanotubes and can be chemically functionalised for physio chemical uses. Mono-, bi-, and tri-layers of h-BN have been synthesized by Siegel *et al.*^3^ from a single precursor, in a chemical vapour deposition system. 2D crystal lattices of h-BN with reliable creation of various defects, for possible use in high quality quantum emitters, were developed using plasma etching techniques by Vogl *et al.*^4^ Different functionalisation methods for fabrication of various BN materials to produce novel properties such as adjustable band gaps, tuneable surface affinities *etc*, are reviewed by Weng *et al.*^5^

Experimental investigations on oxidation of atomically thin BN by Li *et al.*^6^ verified that high-quality single-layer BN can resist oxidation at temperatures above 800 °C, contrasting with oxidation of graphene at 400 °C. Spectroscopic studies with NEXAF technique of hydrogen reaction on oxygen-functionalized h-BN, by Späth *et al.*,^7^ indicated that oxygen atoms are co-adsorbed with hydrogen but no adsorbate–adsorbate interactions were observed. Experiments, by Singh *et al.*,^8^ on exposure of h-BN to oxygen plasma caused a ∼100-fold reduction in electrical resistance and a band gap narrowing to 4.31 eV from predominant doping of oxygen for nitrogen vacancies. Carbon doped BN nanosheets are found to be highly efficient electrocatalysts for ambient nitrogen reduction by Ma *et al.*^9^ A review on spin-gapless semiconductors, by Wang *et al.*,^10^ indicates that C doped and vacancy doped BN nanoribbons are parabolic band spin-gapless semiconductors with potential spintronic applications. Aluminium doping of bulk 2D h-BN, by Legesse *et al.*,^11^ showed diminishing electronic band gap and more thermodynamic stability at higher doping concentrations. Synthesis by *in situ* growth of phosphorus-doped BN on alumina, reported by Lin *et al.*,^12^ led to a robust catalyst for stable ethylbenzene conversion and styrene selectivity.

Computational modelling, such as first principles-based density functional theory (DFT), provides efficient way to investigate insights into the chemical and physical interactions of various defects in real materials. Explorations *via* first-principles calculations of vacancy and substitutional defects in h-BN monolayer, by Azevedo *et al.*,^13^ surmised that the presence of defects can result in the reduction of the energy gap and the possible change of spontaneous magnetisation. Appearance of local magnetic moments was recognized by Fedorov *et al.*^14^ based on DFT investigations on the influence of ordered vacancies on elastic and magnetic properties of graphene-like structures (*e.g.*, BN). Huang *et al.*^15^ find, through first principles calculations, that layer effects are due to local bond lengths been sensitive to changes in their state around charged defect configurations. Abdi *et al.*^16^ from *ab initio* analysis of colour centres in h-BN with various defects, such as vacancies and substitutions, identified spin polarisation channels useful for detecting quantum emitters in h-BN monolayers. Zhao *et al.*^17^ exploring monolayer h-BN with triangular vacancies (B or N vacancies) predicted magnetic and piezoelectric properties, with the triangular holes able to induce spontaneous magnetization in the defect BN system. First-principles calculations by Pan *et al.*^18^ on BN nanoribbons with B or N vacancies predict the presence of spin-polarized semimetals, semiconductors and/or spin gapless semiconductors. Outcomes of alkali atom doping of h-BN and graphene 2D surfaces, from first-principles, by Denis *et al.*^19^ show that increased reactivity of the 2D surfaces strongly depends on the functional group added and the alkali dopant used. The authors concluded that when the alkalis act as reducing agents, then 2D BN is more reactive than graphene while the Li dopant is the more powerful reducing agent.

Studies with larger supercells incorporated multiple vacancies or defect sites (4 or more) on the surface, which meant the BN surface area was dominated by defected sites and had a high defect to surface ratio. This work aims to study, by DFT modelling and calculations derived from the Vienna *ab initio* simulation package (VASP), the physicochemical interactions of single atom reactions with mono-vacancy (B or N vacancies) defected h-BN. A two-dimensional supercell consisting of 128 atoms is chosen for the BN monolayer, allowing the vacancy site to have a smaller surface area relative to the total surface area of the BN layer *i.e*., the vacancy defect will not dominate the BN surface area. Properties such as adsorption energy, Bader charges, adatom displacements, local magnetic moments, possible types of conductivity or semiconductivity are identified from structural relaxation, spin polarisation, density of states (DOS) and energy calculations. A distinctive aspect of this study involves the interaction with flexible movement of all atoms in the simulation *i.e.*, no displacement constrains or symmetry restrictions; an optimised large super cell for the defect BN monolayer; same structural conditions (*i.e.*, same important parameters) in the calculations of properties; eight different atoms from the 1st and 2nd row elements (H, Li, C, O, Al, Si, P, S) of the periodic table are presented together in allowing for comparisons of the calculated properties. Possible chemical reactivity of the adsorbed adatom surfaces with the simple diatomic carbon monoxide (CO) molecule is made to ascertain the thermodynamic feasibility of the interaction.

## 2. Computational details

### 2.1 Modelling and software

The Vienna *ab initio* simulation package (VASP) performed all DFT calculations.^20–22^ Structural optimisation calculations used pseudopotentials for all elements generated from the projector augmented wave (PAW) method^23,24^ with the plane-wave basis energy cut off set to 570 eV. Use of the generalized gradient approximation (GGA) Perdew–Burke–Ernzerhof (PBE)^25^ functionals accounted for the electron exchange–correlation potentials. The Brillouin zone integrations used a *k*-point mesh generated by the Monkhorst–Pack method.^26^ The functionals utilised in the calculations that considered long-range effects from van der Waals interaction used the DFT-D2 corrections of Grimme^27^ by setting the vdW correction flags in the VASP input INCAR files. The total energies on each ion converged to less than 10^−5^ eV with all structures deemed to be fully relaxed until the force constants on every ion were all less than 0.05 eV Å^−1^.

Structural optimization used a 4 × 4 × 1 *k*-point mesh while DOS/Band calculations were performed with a 12 × 12 × 1 *k*-points mesh (72 *k*-point). The BN monolayer is modelled on a 128 atoms (8 × 4) supercell with cell dimensions: *x* = 20.4478 Å, *y* = 17.7084 Å, *z* = 22.1739 Å. A vacuum spacing larger than 20 Å, along the *z* direction, prevents interference between periodic images along the perpendicular direction to the surface keeping unintended interactions negligible while the *x* and *y* directions are repeated periodically. Integration of magnetic moment and local electronic density of state (DOS) were calculated in the PAW sphere. Further details on the implementation of the VASP code are in the ESI Section.†

Formation energy of the defect containing BN surfaces are:

1 *E*_f_ = *E*_defect___surface_ − *E*_hBN_

where, *E*_defect___surface_ = energy of the BN monolayer with 1 N vacancy or 1 B vacancy and *E*_hBN_ = energy of the pristine BN monolayer.

Adsorption energies (*E*_d_) are:

2 *E*_d_ = *E*_defect_surface+atom_ − (*E*_defect___surface_ + *E*_atom_)

where, *E*_defect_surface+atom_ = energy of the defect BN monolayer plus the adsorbed atom, and *E*_atom_ = energy of the adsorbed atom.

Energy minimisation is undertaken, until required accuracy was reached, for reactant atom, defect BN monolayers and the reacted monolayers. For reactions of the defected BN monolayers with the elemental atoms, the energy minimisation was done initially with reactant species located at P (0.5,0.5,0.5) and then at ∼1 Å from the BN layer until successful convergence was achieved. After energy optimisation was reached the final formation and adsorption energies were calculated by eqn (1) and (2) from results of the initial atom at 1 Å from monolayer. Energy minimization for a single atom was done with the same cell configuration and the atom located at P (0.5,0.5,0.5).

### 2.2 h-BN surface with vacancies

**Fig. 1:**
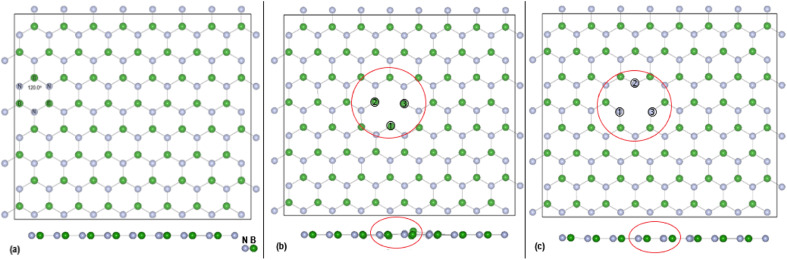
Top view (*z*-axis) of optimised (8 × 4) super cell for: (a) pristine h-BN, (b) V_N_ and (c) V_B_. Also shown is the side (*x*-axis) view and the position of the vacancy site atoms. A red circle bounds the defect area. The sizes of the B and N atoms are proportional to their atomic radii.

DFT calculations, with cell parameters detailed above, for the pristine h-BN monolayer were undertaken until structural optimisation was reached. The mono vacancies in the h-BN structure are then created by removal of one N or B atom from the h-BN 2D layer and structural optimisation undertaken until energy convergence was established. In the subsequent discussions the notations are V_B_, and V_N_ for the B and N vacancy defect h-BN monolayers, respectively. The terminology of the vacancy regions or sites is given as N_v_ (N vacancy sites) and B_v_ (B vacancy sites). The full configuration of the optimised surfaces, viewed along the *z*-axis, of the 128 atoms (8 × 4) super cell for h-BN, V_N_ and V_B_ monolayers are given in Fig. 1 below.

The overall plan of this study through DFT calculations is:

(1) Creation and structural optimisation of the pristine h-BN, V_N_ and V_B_ monolayers (discussed above).

(2) Ascertain thermodynamic viability for reactions of atomic elements from 1st row (H, Li, C, O) and 2nd row (Al, Si, P, S) elemental atoms of the periodic table with the pristine h-BN, V_N_ and V_B_ monolayers. These group of elements consist only of s- and p-orbital atom configurations. The reaction products will be represented as A-V_N_ and A-V_B_ from reactant atoms A = H, Li, C, O, Al, Si, P, S.

Analize charge and structural results from thermodynamically viable reactions (Section 3.1).

(3) Calculate electronic and magnetic properties of various A-V_N_ and A-V_B_ (Section 3.2).

(4) Explore chemical reactivity and thermodynamic viability of reaction by diatomic CO with A-V_N_ and A-V_B_ (Section 3.3).

## 3. Results and analysis

The effectiveness of molecular adsorption/reaction with monolayer defected h-BN surface is measured by determining the adsorption interaction energies calculated by eqn (2).

**Fig. 2:**
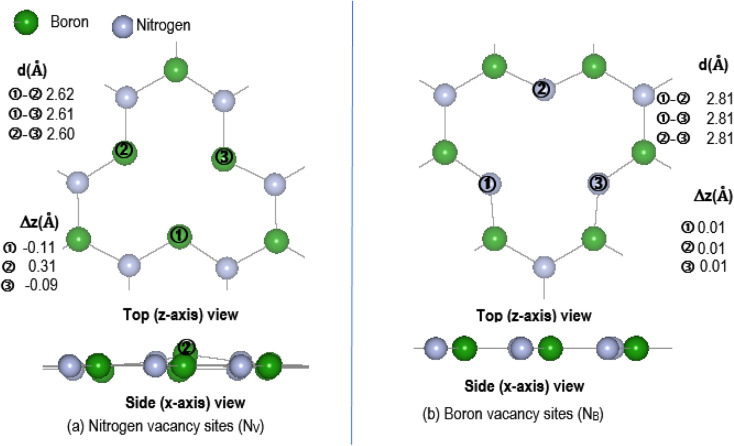
Magnification of vacancy region for pristine V_N_ and V_B_: (a) N_V_ and (b) B_V_. Sites are indicated by ➀, ➁, and ➂. Included are displacements (Δ*z*) of site atoms from the V_N_ and V_B_ planes and site–site distances.

Physical solid-state properties such as, electronic energy band gap, total magnetization charge density, atomic magnetic moments, and energy levels with their orbital contribution to DOS profile are derived by the VASP code. Analysis of these physical properties will complement the chemical interaction results. Bader charge analysis of the charge density data files utilise code from the Henkelman group (University of Texas).^28^ The VESTA^29^ visualization software provided images for the atomic structure, and structural parameters (bond lengths *etc.*). Analysis of the DFT results is focused around the vacancy sites and as such, Fig. 2(a) and (b) are magnifications of the N_V_ and B_V_ regions, derived from Fig. 1(b) and (c). The vacancy site atoms are shown as ➀, ➁, and ➂. Included in the diagrams are: distances *d* (Å) between the vacancy sites (*i.e.*, site–site displacement); displacements (Δ*z*) of the site atoms from the monolayer plane. The vacancy site atoms of the V_B_ monolayer are nitrogen (N) atoms while the vacancy site atoms of V_N_ are boron (B) atoms. The site atoms are in-plane with the V_B_ monolayer while the site ➁ boron atom of the V_N_ layer is 0.31 Å from the monolayer plane.

### 3.1 Atom adsorption with h-BN and defect V_N_ and V_B_ surfaces

Calculations of adsorption energy of single atom A (H, Li, C, O, Al, Si, P, and S) reacting with pristine h-BN, V_N_ and V_B_ monolayers by the energy minimization procedures of Section 2 were undertaken until required accuracy was reached. It is assumed the monatomic H and O reactions can occur with the chosen surfaces inside some type of reactive plasma setup producing the atoms from H_2_ and O_2_ molecules. The configuration of successful adsorption products results with the atom A (H, Li, C, O, Al, Si, P, and S) either in-plane or out of plane to the 2D defected monolayer plane.

#### 3.1.1 Atom-hBN reaction

**Fig. 3:**
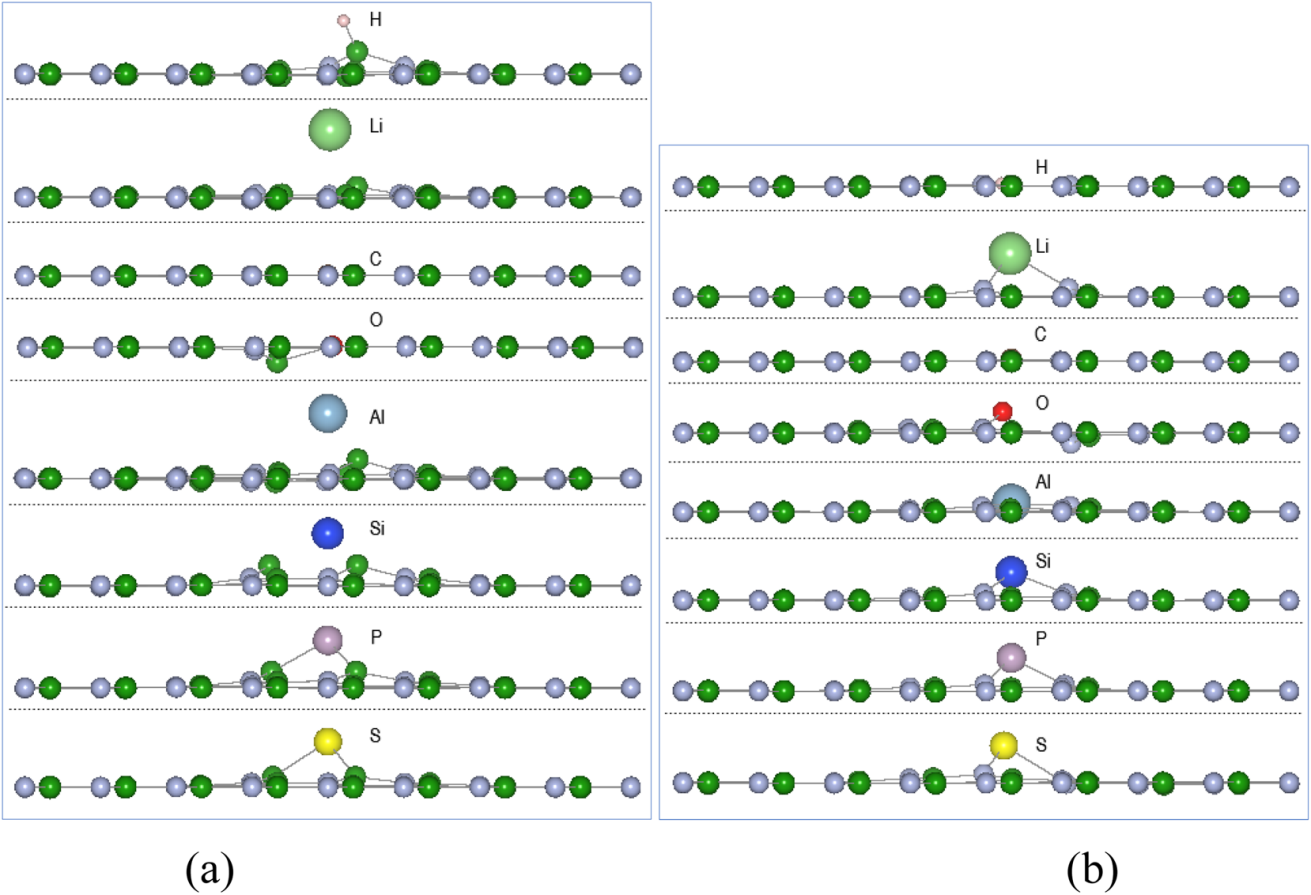
Single atom reaction with V_N_ and V_B_ monolayers, displayed along the *x*-axis: (a) A-V_N_ (b) A-V_B_, with atom A = H, Li, C, O, Al, Si, P, and S.

Reactions of the single atoms H, Li, O, C, Al, Si, P, and S with pristine h-BN result in physisorption with the atom approximately 1.9–3.1 Å from the h-BN plane with reaction energies ranging from 0 to −0.6 eV. An exception is the reaction of single O or C atom resulting in bonds ∼1.6 Å. Sulphur has a more favourable reaction energy of −1.0 eV but is 1.9–2.5 Å from the nearest vacancy site atoms resulting in physisorption process with the monolayer. Overall, the reaction of the chosen single atoms is predicted to have low reaction energies and larger than bonding distances for atom-BN plane distances suggesting the interactions are predominately physisorption processes. The computational comparative study on graphene and BN by Denis and Iribane^34^ found that hydrogenation of BN prefers to occur over B atoms rather than on N as is the also the case here. Reaction of O atom, based on up to 6 × 6 unit cell, resulted in the oxygenated BN sheet having a zig-zag side view configuration. The single atom reactions with h-BN, in this study using a larger cell size, result in a very flat BN sheet with small perturbation at the reacting site, similar to Fig. 3 (only all atoms are above the BN sheet).

#### 3.1.2 Atom-V_N_ and atom-V_B_ reactions

Fig. 3 displays the configuration of products from the reaction of chosen atoms with the defect boron nitride surfaces, shown along the *x*-axis of the cell. The displayed size of the atoms is proportional to their ionic radii. Table 1a and b list details of adsorption energy, atom displacement, and atom-site nearest neighbour distances for A = H, Li, C, O, Al, Si, P, and S. As the vacancy sites are either N or B atoms, the bond length between atom A and vacancy sites in terms of the atomic radii of the atoms involved are: A–B = *r*_A_ + *r*_B_ and A–N = *r*_A_ + *r*_N_

Adsorption and structural results for A-V_N_ and A-V_B_^a^

**Table:** 

(a) A-V_N_						
A	*E*_d_ (eV)	Δ*z*	A-➀	A-➁	A-➂	*r*_A_ + *r*_B_
H	−4.1	1.55	(2.62)	1.20	(2.61)	1.27
Li	−1.2	1.97	2.37	2.30	2.36	2.38
C	−11.7	**0.02**	1.54	1.54	1.54	1.58
O	−9.2	**0.04**	1.69	1.47	1.47	1.55
Al	−2.7	1.90	2.28	2.35	2.12	2.24
Si	−5.5	1.48	1.95	1.95	1.95	1.99
P	−8.0	1.35	1.88	1.88	1.88	1.91
S	−6.3	1.31	1.90	1.90	1.90	1.85

a Adsorption energy *E*_d_; displacement (Δ*z*) of adatom (A) from the V_N_ or V_B_ plane; ➀, ➁, and ➂ are the vacancy sites: B atoms in A-V_N_; N atoms in A-V_B_; A-➀, A-➁, and A-➂ are nearest neighbour distances; *r*_A,_*r*_B_, and *r*_N_ = adatom (A), boron and nitrogen atomic radii respectively; Fig. 2 indicates the positioning of the vacancy sites; all displacements/lengths in Å units.

**Table:** 

(b) A-V_B_						
A	*E*_d_ (eV)	Δ*z*	A-➀	A-➁	A-➂	*r*_A_ + *r*_N_
H	−5.1	**0.11**	1.03	(2.06)	(2.06)	1.2
Li	−4.8	1.26	1.92	1.98	1.92	2.31
C	−14.5	**0.08**	1.43	1.43	1.43	1.51
O	−7.1	0.62	1.48	(2.20)	1.48	1.48
Al	−10.8	**0.26**	1.72	1.72	1.72	2.17
Si	−12.0	0.82	1.73	1.73	1.73	1.92
P	−12.5	0.95	1.74	1.74	1.74	1.84
S	−7.1	1.08	1.70	2.14	1.70	1.78

Where *r*_A_ = atomic radii of H, Li, C, O, Al, Si, P, or S and *r*_B_, *r*_N_ = atomic radii of B and N respectively.

The last column of Table 1 lists the atom-vacancy atom bond lengths derived from the atomic radii. If at least one of the A-➀, A-➁ or A-➂ distances from Table 1 approach to a distance approximately equal to or less than the sum of the atomic radii, then can consider that there is a definite bonding action between the adatom and the defect monolayer.

The adsorption energies from Table 1 indicate thermodynamic feasibility and highly exothermic atom interaction with the defect surfaces with adsorption energies for V_B_ > V_N_. Of the 16 products, four (C-V_N_, O-V_N_, H-V_B_, and C-V_B_) have the adatom in-plane (*i.e.*, < 0.1 Å from plane) and symmetrically centred at the vacancy. The other thirteen products have the adsorbed atom in the range of 0.26–1.97 Å from the surface plane and located in the vacancy area. Carbon and oxygen adsorption with the V_N_ surface cause the initially displaced site ➁ atom to shift in-plane, while the adsorption of the other atoms have kept the boron site ➁ atom within 0.1–0.4 Å from the V_N_ plane. The oxygen adsorption with the V_N_ surface also causes the site ➀ atom to shift ∼0.1 Å from the V_N_ plane. The H atom, in H-V_B_, binds with a B atom in a similar manner with the pristine h-BN reaction but is ∼0.4 Å closer to the BN sheet while with V_B_ the H bonds with N atoms in-plane to the BN sheet.

Comparing the atom(A)-site distances with the atomic radii distances listed in Table 1, it is deduced that the reaction process is chemisorption with the adatom chemically bonding with either a B or N site atom. Furthermore, the structural integrity of the monolayer is retained and with very minor site–site distance deviations from the pristine V_N_ and V_B_ monolayers. Vacancy defected h-BN or single atom doping of the defected BN do not lead to structural instability of the BN nanosheet. Even so, from the binding energy results it is seen that V_N_ is the least energetically favourable defect. C-V_N_ and C-V_B_, with the highest binding energies (*E*_b_ = −11.7 and −14.5 eV), are comparable to the hydrogenated BN structures such as B_27_N_26_H_18_C and B_26_N_27_H_18_C (*E*_b_ = 17.1 eV), studied by Anota *et.al.*,^35^ where C is substituted in-plane in the N or B vacancy.

The cohesion energy (*E*_coh_) related to the binding/adsorption energy, is increased in the A-V_B_ structures with *E*_coh_ for 2nd row atoms (Al, Si, P, S) >*E*_coh_ 1st row atoms (H, Li, O). A similar trend is seen with A-V_N_ structures. The atoms that are bonded in-plane (C, O, H) of the BN sheet have higher cohesion energies than those above the BN sheet *i.e.*, substitutional defects in BN sheets may have improved cohesion energies than externally bonding to the BN sheet.

The Bader charge differences listed in Table 2 show that for A-V_B_ products the surface vacancy site N atoms transfer charge to the adatom, with the exception of the O adatom which transfers charge to the N atoms. In contrast, the adatom in the A-V_B_ products transfers charge to the vacancy site B atoms, except for the Al and Si adatoms in which the charge transfer is from the vacancy site B atoms. The H and Li adatoms have their total valency charge transferred either to or from the vacancy site atoms of V_N_ and V_B_. Interestingly, the charge transfer from the BN to the Li atom of ∼0.99*e*^−^ is in contrast to the cases of Li donating charge to functionalised BN surfaces as found by Denis *et al.*^19^ The Al adatom has its total valency charge transferred from the vacancy site atoms of both V_N_ and V_B_. All A-V_N_ and A-V_B_ are polar as is generally found for non-stoichiometric surfaces. The A-V_N_ surfaces are positively polarised while the A-V_B_ surfaces are negatively polarised, as can be deduced from Table 2. The overall polarity, defined as the charge balance of the adatom and the 3 vacancy sites, in absolute values, of the A-V_N_ surfaces compared to the A-V_B_ surfaces is larger for all adatoms except for Li and S adatom. The order of polarity for the surfaces in terms of the adsorbed adatom is: C > (Si ≅ P) > (H, O, Al) > Li for the A-V_N_ products and C > (Si ≅ P ≅ S) > (O ≅ Al) > (H, Li) for the A-V_B_ products.

**Table:** Bader charge differences of adatoms and vacancy sites ➀, ➁, and ➂ for A-V_N_ and A-V_B_.^a^

A	Q(A)	Q(➀)	Q(➁)	Q(➂)
**(a) A-V**_**N**_				
H	1.05	0.193	−0.569	0.158
Li	−0.991	0.408	0.383	0.361
C	3.24	−0.668	−0.569	−0.691
O	1.99	−0.027	−0.569	−0.691
Al	−3.00	1.77	0.374	1.44
Si	−3.87	1.62	1.71	1.62
P	0.396	0.157	0.278	0.178
S	2.24	−0.668	−0.569	−0.691

**(b) A-V**_**B**_				
H	−1.00	0.801	−0.052	−0.034
Li	−0.988	0.235	0.129	0.262
C	−2.61	0.609	0.557	0.628
O	0.274	−0.412	−0.028	−0.404
Al	−3.00	0.791	0.791	0.832
Si	−3.13	0.830	0.825	0.874
P	−3.23	0.849	0.843	0.901
S	−2.52	0.827	0.207	0.869

a Bader charge difference, *Q*, is in |*e*^−^| units; vacancy sites ➀, ➁, and ➂ are: B atoms in A-V_N_; N atoms in A-V_B_; Fig. 2 indicates the positioning of the vacancy sites.

Overall, the production of vacancy sites leads to a more reactive surface with reactants interacting and bonding with the vacancy site atoms leading to a range of charge transfer magnitudes to or from the site atoms by the reactant atom and positive or negative charged polar surfaces.

### 3.2 Electronic and magnetic properties of mono-atomic adsorbed V_N_ and V_B_ surfaces

The magnetic and electronic properties of materials can be described by important solid-state parameters such as: *μ*_τ_, *μ*_atom_, and *μ*(➀, ➁, ➂) for the total, atom, and vacancy site atom magnetic moments respectively; the valence band maximum (VBM); conduction band minimum (CBM); Fermi energy (*E*_F_); and electronic energy band gap (*E*_g_). Energy bands with nonzero occupancy were considered to be occupied in the calculation of *E*_g_, with its magnitude calculated as: *E*_g_ = |CBM − VBM| (eV). The top of the valence band, VBM, can be a minority or majority spin state with the top of the conduction band differing for majority and minority spin states. Energy levels in the forbidden band gap region could be due to energy states from the adsorbed atom and can give rise to extrinsic semiconductivity. An important feature of semiconductors has the Fermi energy inside the band gap (*E*_F_ > VBM) but it's also possible for the material to have conductive behaviour if *E*_F_ ≤ VBM. Anisotropic properties of h-BN, arising from the induced ionicity and electron transfer from boron to nitrogen, make it an electric insulator with a band gap up to 6 eV.^1^ The pristine h-BN monolayer, considered here, has a calculated 4.54 eV electronic energy band gap, while calculations for the defected pristine monolayers and the reaction products show a narrowing of the energy gap (see Table 3).

**Table:** Energy band gaps of A-V_N_ and A-V_B_. Local magnetic moments for monolayer, adatom (A), and vacancy sites ➀, ➁, and ➂^a^

A	*E*_g_	*μ*_τ_	*μ*_A_	*μ*➀	*μ*➁	*μ*➂	Notes
**(a) A-V**_**N**_							
H	3.56	0.00	0.00	0.00	0.00	0.00	i
Li	0.36	−0.97	−0.12	−0.13	−0.32	−0.13	c; e; i
C	4.28	0.54	0.314	0.01	0.01	0.01	d; i
O	0.98	−0.45	−0.03	−0.31	−0.00	−0.00	d; i
Al	1.23	0.00	0.00	0.00	0.00	0.00	d
Si	3.63	0.39	0.24	0.02	0.02	0.02	d; i
P	4.14	0.00	0.00	0.00	0.00	0.00	i
S	0.77	−0.42	−0.05	−0.09	−0.09	−0.09	c, e; i
Pristine^b^	1.53	0.49	—	0.07	0.24	0.06	

**(b) A-V**_**B**_							
H	4.20	1.49	0.01	0.05	0.56	0.56	c; d; i
Li	3.77	1.58	0.02	0.39	0.39	0.45	c; d; i
C	0.43	0.61	0.38	0.01	0.01	0.01	c; d; d
O	3.33	0.80	0.17	0.00	0.00	0.53	e; i
Al	4.52	0.00	0.00	0.00	0.00	0.00	i
Si	2.97	0.46	0.25	0.06	0.06	0.06	e; i
P	3.65	0.00	0.00	0.00	0.00	0.00	i
S	2.08	0.70	0.33	0.01	0.01	0.26	e; i
Pristine^b^	4.28	2.23	—	0.55	0.55	0.55	

a Energy band gap = *E*_g_ (eV); *μ*_τ_ = total magnetic moment, *μ*_A_ = adatom (A) local magnetic moment; *μ*➀, *μ*➁, *μ*➂ = local magnetic moments of vacancy site atoms; all moments in *μ*_B_ units. Vacancy sites ➀, ➁, and ➂ are: B atoms in A-V_N_; N atoms in A-V_B_.

b Pristine V_B_/V_N_ from ref. 31.

c VBM > *E*_F_ – possible conductivity.

d VBM is a minority spin state.

e VBM is a majority spin state; i, d specify indirect or direct band gap respectively.

**Fig. 4:**
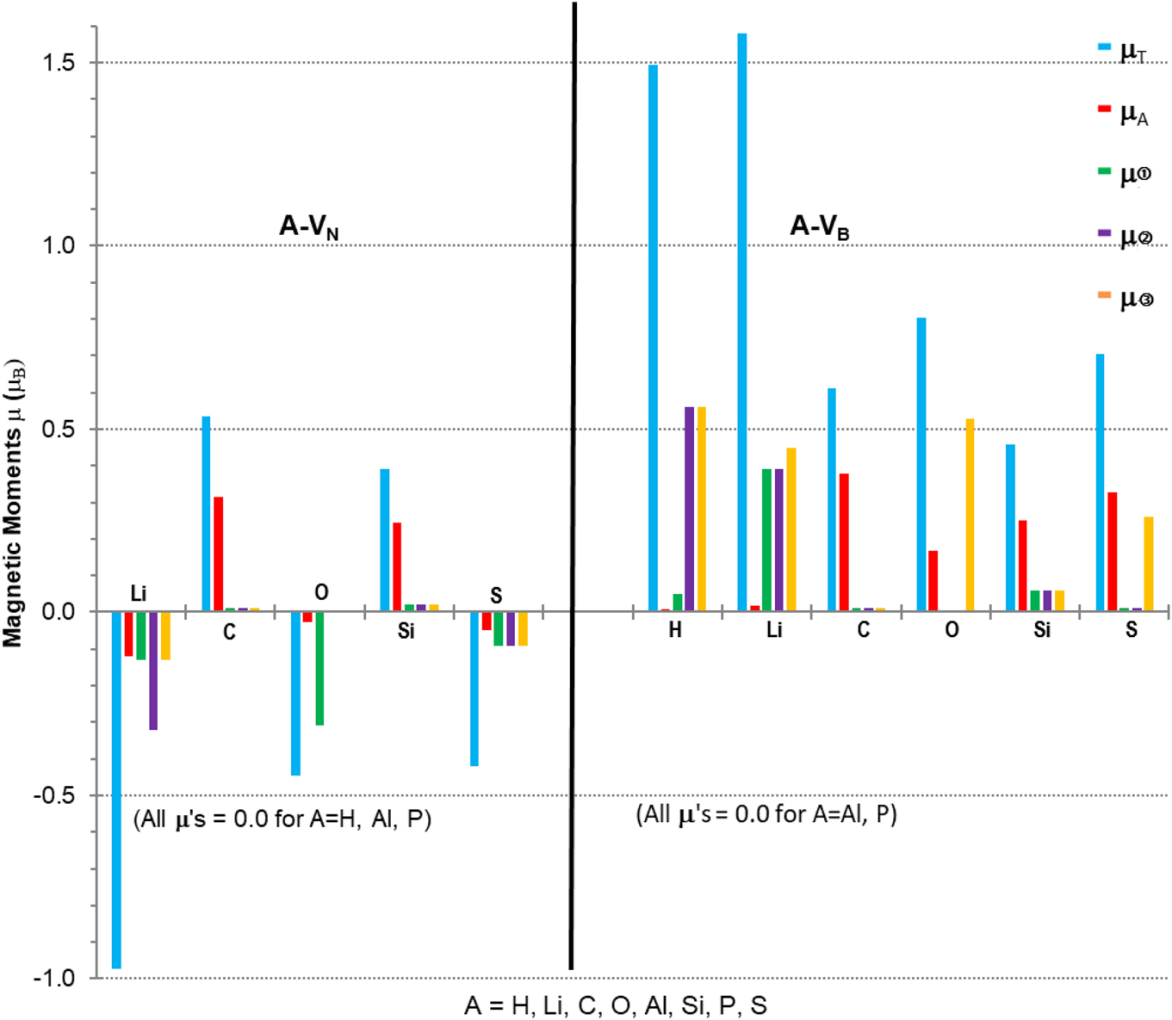
Magnetic moments for A-V_N_ and A-V_B_ (A = H, Li, C, O, Al, Si, P, S). *μ*_τ_ = total spin polarisation; *μ*_A_ = local adatom atomic magnetic moment; *μ*➀, *μ*➁, *μ*➂ = vacancy site atom magnetic moments. The vacancy sites for A-V_N_ are B atoms while for A-V_B_ are N atoms.

Calculations to obtain magnetic moments and DOS data were performed with a 12× 12 × 1 *k*-points mesh (72 *k*-point) using the computational method discussed in Section 2. Programs from the VST tools^28^ were used to calculate the energy band gap, *E*_g_, VBM, CBM, types of spin states and band gaps. Values of the energy gap and magnetic moment parameters for the monolayers with different atomic adsorbents are presented in Table 2 with the last column indicating possibilities for type of semiconductivity, conductivity, minority or majority spin state of VBM. Fig. 4 is a plot of the non-zero magnetic moments (from Table 2), in a bar graph style for visual comparison of the magnetic moments that clearly indicates the direction of spin polarisation.

The A-V_N_ products, with non-zero *μ*_T_, have approximately total magnetic moments similar to pristine V_N_, except for the Li adsorbent material which almost doubles it. A significant change is in the total spin polarization for the structures with Li, O, or S adsorbents which have a spin down (s↓) state. The product A-V_B_ surfaces show the total spin polarisation is in the spin up (s↑) state.

The adatom magnetic moment contribution to the total magnetic moment of the material varies from 1% to 62% with the bigger contributions (>50%) from the C, Si and S adatoms. All products, except for the O containing products and Li-V_B_, with non-zero total magnetic moment have spin polarization in the *E*_F_ region ranging from 0.5 to 1.58 eV (see Table 3). The A-V_B_ products with non-zero magnetic moments have larger vacancy site atom contribution to *μ*_T_ from the N atoms while the A-V_N_ products have smaller contributions from the B atoms to *μ*_T_. The pristine V_N_ and V_B_ have total and vacancy site magnetic moments, listed in Table 3, but reaction with H (in V_N_), Al or P atoms completely supress spin polarization resulting in zero magnetic moments. The chemical atom interaction from single Li, O, and S atoms with V_N_ is the cause of the magnetic moments in A-V_N_ as the spin polarisation direction of the total moments is altered and total monolayer moment *μ*_T_ is larger than the sum of the atom and vacancy moments. A more subtle interaction occurs with the unshared 2p/3p *e*^−^ of the C and S atoms with V_N_ as there is a decrease in the vacancy sites spin polarisation (especially with site ➁) and with the values of *μ*_A_ and *m*_T_ suggest that magnetic moments arise from both the atom and the vacancy sites. The magnetic moments magnetic moments of the H and Li atom in H-V_B_ and Li-V_B_ are due to the vacancy sites. As with the A-V_N_ case a subtle interaction occurs with the unshared 2p/3p *e*^−^ of the C, O, Si and S atoms with the V_B_ layer suggesting magnetic moments come from both the adatom and the vacancy sites. Hydrogenated armchair BN nanosheets with homonuclear boron bonds, studied by Anota,^36^ with excess B atoms (*e.g*., B_33_N_21_H_18_) showed magnetic moments of 2 *μ*_B_ that is due to boron hybridization with the N in the sheet. The vacancy formed h-BN sheets with only normal B–N bonds had *μ* of 0.49 and 2.3 *μ*_B_ while the A-V_N_ and A-V_B_ surfaces ranged from −0.97 to 1.58 *μ*_B_.

**Fig. 5:**
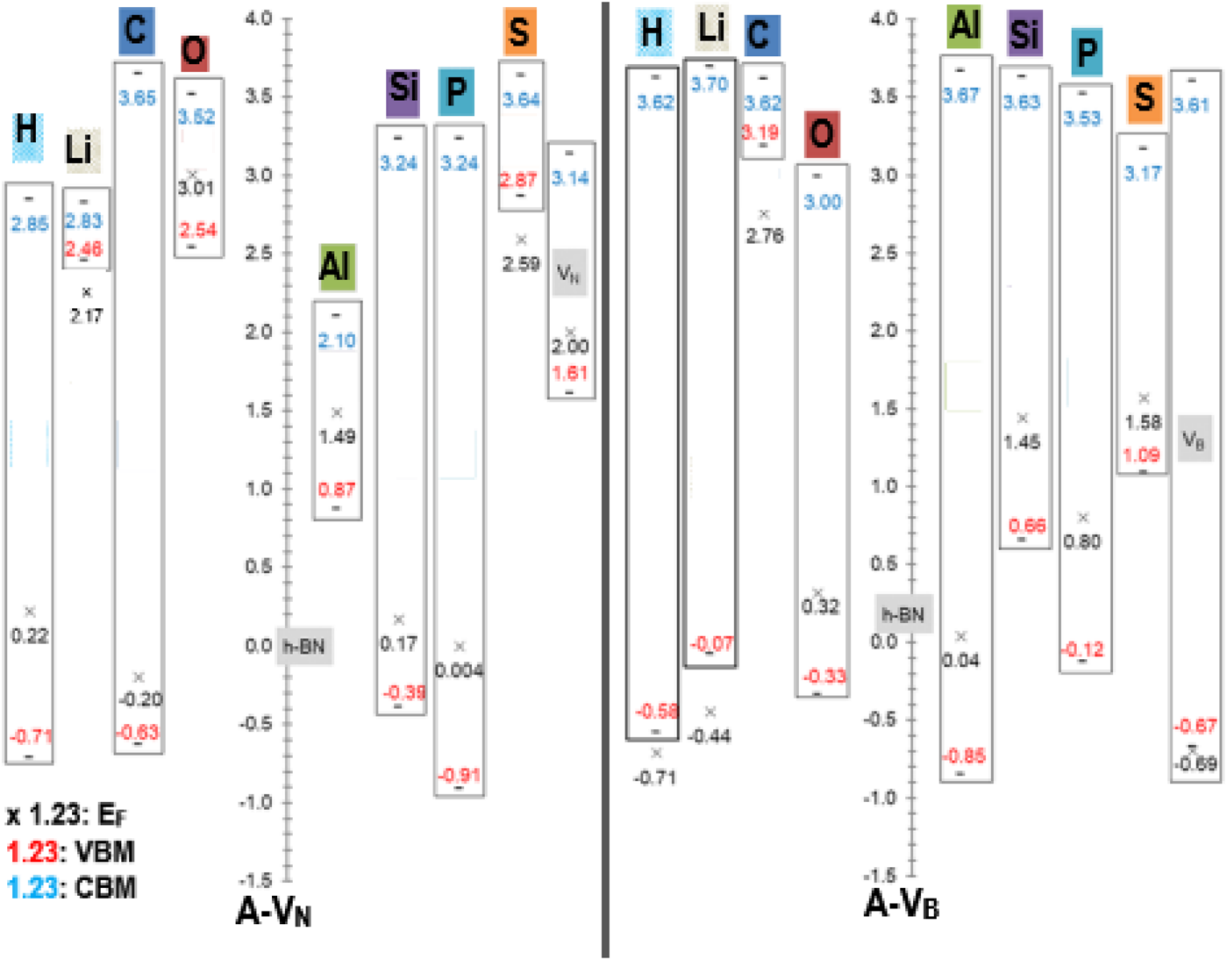
Fermi energy levels *E*_F_, VBM, and CBM with respect to pristine h-BN (*E*_F_ = −3.686 eV), for A-V_N_ and A-V_B_ (A = H, Li, C, O, Al, Si, P, and S). All energy units are in (eV).

An equally useful visual comparison of the results from the DOS calculations, using a modified column style design, are plots of the valence and conduction band edges, *E*_F_ levels and band gaps as displayed in Fig. 5. The plots are to scale and indicate all energy values, with respect to the pristine h-BN monolayer. This is done as h-BN is the initial monolayer from which the N or B vacancies were produced. The zero point corresponds to the pristine h-BN *E*_F_ level (−3.686 eV). The band gap *E*_g_ is calculated as |CBM − VBM| with the length of the rectangles giving a pictorial view of the band gap magnitude. All energy units are in (eV) with values of *E*_F_ < VBM located outside the rectangle. Data from the pristine unreacted V_N_ and V_B_ monolayers, are also incorporated in the figure and indicated with the grey highlight colour.

The raw energy values for each product can be calculated from information given in Fig. 5 by adding (−3.686) to the values displayed in the figure. As an example, raw energy values for O-V_N_: *E*_F_ is −3.686 + 3.01 = −0.676 eV; VBM is −3.686 + 2.54 = −1.146 eV; CBM is −3.686 + 3.52 = −0.166 eV. The band gap values also correspond with the values tabulated in Table 2.

An example of the usefulness of Fig. 5 may arise in investigations of using material with adhered A-V_N_ and/or A-V_B_ layers as one electrode of a two-electrode setup in contact with a redox electrolyte for catalytic, or photo electrochemical applications. Here, the position of the *E*_F_, VBM and CBM energy levels (of liquid and electrodes) and *E*_Redox_ of the composite system (solid electrodes + liquid) will dictate the outcome as it will depend on shifts and differences in magnitudes of the energy levels as redox reactions occur and/or the composite material is illuminated by some light source. The alkali atom doping of 5 × 5 BN sheet, studied by Denis *et al.*,^19^ found *E*_g_ values of 1.78/4.45 eV for Li dopant and 4.45/1.0 eV for H dopant where the 2 values represent spin ↑/spin↓ gap.

The full spin polarised DOS (SDOS) plots for pristine V_N,_ V_B_, and all the A-V_N_, A-V_B_ products, from the spin polarised calculations, are presented in Fig. S1 to S10 of the ESI.† All SDOS plots are with respect to the *E*_F_ level of the material *i.e.*, the zero point is equal to *E*_F_ (eV). The full plots indicate the VBM, CBM levels, s- and p- orbital contribution from the B, N, and adatom. To enhance the visibility of the adatom contribution to the full SDOS spectrum, the DOS magnitudes of the adatom have been multiplied by a factor of ×10 to ×50 and are shown in the graph's legend. Included are magnification plots of the *E*_F_ region specifically displaying any s, p, p_*x*_, p_*y*_, and p_*z*_ orbital contribution from the adatom.

Energy band levels and spin states, taken from the SDOS plots, for the adatom with orbital contributions, in the *E*_F_ region are summarised in Table 4. Any orbital (*i.e.*, s, p_*x*_, p_*y*_, or p_*z*_) contributions from the adatom are given in blue with spin states indicated by the red arrows. Included in the table are any difference (or widths) in the spin polarisation (Δ*E*_s_). Analysis of all DOS plots reveals that all of the adatoms, except for Li in Li-V_B_, have orbital contributions near the vicinity of the *E*_F_ level.

**Table:** A summary of orbital contribution from the adatom in A-V_N_ and A-V_B_ (A = H, Li, C, O, Al, Si, P, S) in the vicinity of *E*_F_

A		*E* a	Δ*E*_s_^b^
**(a) 1st row atoms A = H, Li, C, and O**			
H	V_N_	−0.92↑↓ s	—
	V_B_	−1.39↑/+0.13↓ s	**1.52**
Li	V_N_	−0.28↓/+0.32↑ s	**0.6**
	V_B_	d	
C	V_N_	−0.42↑/+0.43↓ p_*z*_	**0.85**
		−1.84↑/−1.72↓ p_*z*_^c^	0.12
	V_B_	−0.43↑/+0.47↓ p_*z*_	**0.90**
O	V_N_	−0.46↓ p_*y*_ p_*z*_	—
		+0.58↑/+0.54↓ p_*z*_	0.04
	V_B_	−0.66↑/−0.92↓ p_*y*_ p_*z*_	0.26
		+0.66↓ p_*y*_ p_*z*_	

**(b) 2nd row atoms A = Al, Si, P, and S**			
Al	V_N_	−0.61↑↓ p_*x*_ p_*y*_ p_*z*_	—
		+0.63↑↓ p_*x*_ p_*y*_	—
		+0.99↑↓ p_*z*_^c^	—
	V_B_	−2.18 p_*x*_ p_*y*_^c^	
Si	V_N_	−0.59↑/+0.55↓ p_*z*_	**1.14**
		−0.67↑/−0.55↓ p_*x*_ p_*y*_	0.12
	V_B_	−0.78↑/+0.80↓ p_*z*_	**1.58**
P	V_N_	−1.10↑↓ p_*z*_^c^	—
		−1.28↑↓ p_*x*_ p_*y*_^c^	—
		+3.24 ↑↓ p_*z*_	—
	V_B_	−0.92↑↓ s p_*z*_	—
		+2.78↑↓ p_*x*_ p_*y*_	—
S	V_N_	−0.25↓/+0.27↑ p	**0.2**
	V_B_	−0.47↑/+0.49↓ p_*y*_	**0.96**
		+1.61↑/+1.71↓ p_*x*_ p_*y*_	**1.0**

a *E* (eV) is given with respect to the *E*_F_ levels of A-V_N_ or A-V_B_ (*E*_F_ is the zero point), *i.e.*, unmodified energy level = *E* (from this table) + *E*_F_ (for A-V_N_ or A-V_B_ deduced from Fig. 5).

b Difference (or width) in the spin polarisation Δ*E*_s_ (eV *c*–*e* have been displayed correctly in Table 4.). The bold indicates spin polarization across the *E*_F_ level.

c Not in VBM/*E*_F_/CBM region.

d No adatom contribution in the vicinity of *E*_F_.

Table 4 shows that half of the products have a spin polarization at the *E*_F_ level of width between 0.5–1.58 eV. Energy levels further from *E*_F_ with spin polarization have smaller widths between 0.1–0.12 eV. For the p block adatoms (C, O, Al, Si, P, and S) the main orbital contribution from the adsorbate near *E*_F_ is from the p_*z*_ orbitals with a spin polarization. Inspection of all the SDOS plots reveal a decrease in the density of states at the Fermi level results from a state crossing the Fermi level and a decrease of that state. The O-V_N_ and O-V_B_ products are different in that they have no spin polarization at the *E*_F_ region and also non-zero magnetic moments.

**Fig. 6:**
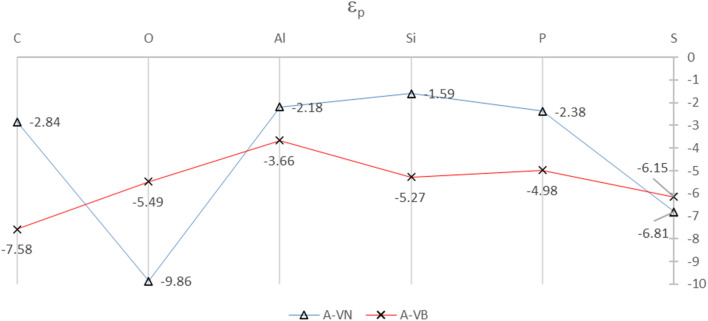
Orbital centers, *ε*_p_, of 2p and 3p orbital bands for the adatoms for the A-V_N_ and A-V_B_ products. The full colour lines are drawn as a guide to the eye. All units are in eV.

The SDOS plots indicate that the 2p and 3p valency orbitals have a wide distribution in the valence band up to the Fermi energy level. It will be interesting to derive the average energy for the orbital center (*ε*_p_) of the adatom 2p or 3p valency orbitals. The orbital center of the adatom is calculated from the projected density of states of a given adatom in an energy interval that is basically the widths of the valence band up to *E*_F_. The idea is from the d-band center (*ε*_d_) that is widely used to interpret and predict interaction strengths for many different adsorbates and metals for metal surface activity such as catalytic activity. It is a useful measure of the position of the d-states in interactions that involve atoms with d-orbitals.^32,33^ The orbital centers for the adsorbates, in the A-V_N_ and A-V_B_ products, were calculated from a total energy width up to the *E*_F_ level ranging from −9.5 eV to −13.5 eV, by the dosanalyze.pl Perl routine from the VST tools packages.^28^Fig. 6 displays the calculated orbital centers of the adatoms in the A-V_N_ and A-V_B_ products with details of the valence bandwidth and standard deviation errors included in Fig. S11 and S12 of the ESI.†

A general trend in the average energy, *ε*_p_ of the p orbital bands from Fig. 6 is that with the A-V_N_ products, except for O-V_N_ and S-V_N_, the availability (occupation) of p electrons for interaction are mainly from localised states near *E*_F_. This trend follows the general rule that electrons at the Fermi level are the most energetically available electrons for chemical reaction. In contrast the A-V_B_ products, O-V_N_ and S-V_N_, the interaction of the adsorbate atoms and the BN surface involves the availability (occupation) of adsorbate p electrons from states in the entire p-band. The trend of the orbital centers is similar to the trend in interaction strength of adsorption for both A-V_N_ and A-V_B_, with slight variation for the C and O adsorbates, suggesting some correlation between interaction strength and the p-center.

### 3.3 Carbon monoxide (CO) reaction with A-V_N_ and A-V_B_ surfaces

An exploration of the chemical reactivity of the surfaces, from Section 3.2, with the simple CO diatomic molecule is made to ascertain the thermodynamic feasibility of the reaction. Adsorption of CO molecule on Al doped h-BN (notated as Al-BN), studied by Zhang *et al.*^37^ showed strong interaction with Al-BN, with a resulting Al–CO bond of 2.12 (Å) and *E*_b_ of −0.75 (eV) which are similar to calculated Al-(CO-V_N_) values of Table 5 below. Adsorption of CO molecule on Si doped BN nanotubes (termed BNNT) were theoretically studied by Wang and Zhang.^38^ The Si replaced a B or N atom (termed Si_N_-BNNT-CO and Si_B_-BNNT-CO) at two different configurations in the nanotube. The results for Si_N_-BNNT-CO had the CO molecule at 1.922 Å and 1.749 Å from the BNNT while for Si_B_-BNNT-CO the CO was 1.96 Å and 3.907 Å from the BNNT and reaction energies in the range −0.038 to −0.756 (eV). Tabtimsai *et al.*,^39^ theoretically investigated CO adsorption on C doped pristine and Stone–Wales defected single wall BNNT with various configurations of the doping. The calculations show the CO molecule at a range of 2.393 Å to 3.249 Å from the BNNT.

**Table:** Reaction of CO molecule with: (a) A-V_N_ and (b) A-V_B_ surfaces^a^

A	*E*_b_ (eV)	C–O (Å)	A–CO (Å)	*r*_A_ + *r*_C_ (Å)
**(a) CO-(A-V**_**N**_ **)**				
Li	−1.1	1.15	2.1	2.3
C	−0.1	1.19	1.5	1.5
O	−1.1	1.19	1.7^c^	1.6^c^
Al	−2.1	1.16	1.9	2.2

**(b) CO-(A-V**_**B**_ **)**				
H	−4.1	1.21	1.5^b^	1.5^b^
Li	−0.1	1.13	2.2	2.3
Al	−0.1	1.13	2.2	2.2
Si	−0.1	1.18	1.8	2.0

a *r*_A_ = adatom (A = H, Li, C, O, or Si) atomic radii and *r*_C_ = carbon atom radius; *E*_b_ = reaction energy (eV).

b CO bonds to site ➂ *i.e*., C–N bond; H bonds to a N atom from BN matrix.

c CO bonds to site ➂ *i.e*., C–B bond; O-site distances, of the adatom, are unchanged and in-plane.

In a previous DFT investigation on molecular interactions with defected h-BN, by the current authors,^31^ it was also determined that it is thermodynamically feasible for the CO molecule to react with the pristine V_N_ and V_B_ surfaces. The DFT results indicated that the CO molecule bonded to the V_B_ surface *via* the C atom and N sites of the surface in a vertical configuration. However, bonding of the CO molecule with the V_N_ surface occurred by the O atom bonding with the B sites or the C and O atoms bonding with the B sites, depending on whether the CO molecular axis was vertically or horizontally aligned to the BN plane in the interaction.

**Fig. 7:**
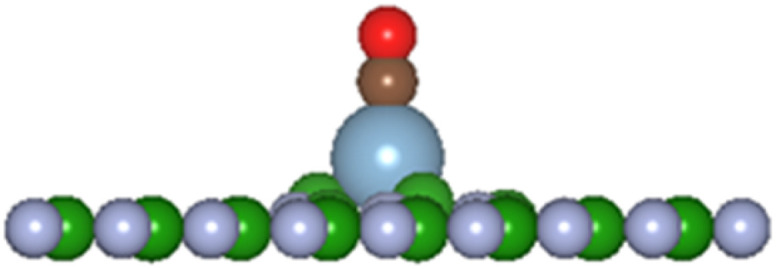
General product geometry of CO bonded to the adatom of the A-V_N_ and A-V_B_ surfaces. 
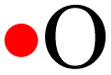

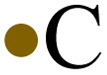

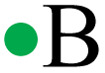

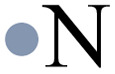
 and 
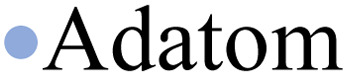
 (shown is the Al adatom).

In the present investigation the V_N_ and V_B_ surfaces have an adatom in the vacancy space and as such interaction of other molecular species will either chemically bond with the adatom, be physisorbed, or break up the adatom-surface configuration. The DFT results predict the three possibilities can occur with diatomic CO reacting with the adatom bonded to the BN surface. Table 5 lists results of the reaction energies (*E*_b_), C–O lengths, and A–CO distances (A = H, Li, C, O, Al, Si, P, S) for successful adatom chemical bonding reactions. The overall product geometry for successful A–CO bonding is indicated in Fig. 7, and shown for the Al adatom product. The adatom (A) bonds to the carbon of the CO molecule with the (A–C–O) component perpendicular to the BN surface plane *i.e.*, CO pillars. In contrast, the H-V_B_ product has the H atom bonding to a N atom from the BN surface while the CO molecule bonds to the N atom of site ➂.

Structural energy minimisation of CO reacting with H-V_N_, S-V_N_, and P-V_B_ could not reach convergence and with the CO molecule >3 Å from the surface resulting in physisorption process. CO interaction with P-V_N_ results with P adatom displaced closer to the BN surface plane, but with CO approximately 5 Å from surface. The reaction of CO with Si-V_N_ results in the Si adatom detaching from the BN surface. The CO reaction with C-V_B_ and O-V_B_ products have the CO molecular axis parallel with the surface and with the molecule >2.5 Å from the surface, suggesting a predominant physisorption process. The CO-(C-V_N_) distance is much shorter than distances listed for the CO interactions with BNNT studied by.^39^

An interesting outcome is from the reaction of CO with S-V_N_ which results in S–C and O.

*i.e.*, CO + S-V_N_ ⇒ S–C + O + [V_N_ surface].

Since the calculated S–C distance (1.89 Å) is not very different from the bond length derived by the atomic radii (1.81 Å) can claim a S–C bond is formed with the molecule stretched and with an activated O atom in an excited state. It may be possible to use the S–C product to form a carbon-sulphur polymer (CS), as theoretically hypothesised by Genin and Hoffmann^30^ by the extended Hückel method, or use the activated O atom for physiochemical interactions.

## 4. Summary/discussion

In summary, it's thermodynamic feasible to produce h-BN mono layers with single a vacancy defect (N or B) and have elemental atoms (A) from the 1st and 2nd row elements (A = H, Li, C, O, Al, Si, P, and S) react, with high negative binding/adsorption energies, and bond with the defected h-BN surface. The Bader charge differences from/to the adatom are of significant magnitude with significant chemical bonding of adatom-surface atom plus stability of the whole BN layer to warrant investigation of the A-V_N_ and A-V_B_ products for further chemical synthesis applications.

The A-V_N_ and A-V_B_ material show a range of solid-state electronic properties, derived from spin polarised calculations and resulting DOS plot: total magnetic moments for the materials with significant contribution from the adatom; lowering of the energy band gap; adatom contribution to the Fermi energy level, *E*_F_; significant spin polarisation in the *E*_F_ region, from adatom. These properties, arise from directional p-orbital (s-orbital for H and Li atom) interactions with the adatoms towards the surface. Calculation of p-band centers show the adsorbates from the A-V_N_ products have most of their available p electrons from localised states near *E*_F_ while the A-V_B_ products have available p electrons energies further away from *E*_F_ (>−3.5 eV). Predicted *E*_F_, VBM and CBM values may be of use in investigating systems containing the A-V_N_ and A-V_B_ monolayer material interacting with other solid or liquid surfaces, *e.g.*: A-V_N_/A-V_B_-metal surfaces; A-V_N_/A-V_B_-electrolyte-metal interaction, possible for catalytic, or photo electrochemical applications.

The DFT exploration on diatomic molecule reaction with the A-V_N_ and A-V_B_ products indicate chemical reactivity of CO with some of the products resulting in ‘pillars’ of CO attached to the adatom of the surfaces. Other predicted outcomes, from the CO reactions, include detachment of the adsorbed atom (*e.g.*, Si), discrete A–C species (*e.g.*, S–C) and excited O atom from the C–O molecule break up.

## 5. Conclusions

This comprehensive DFT-driven work has thoroughly investigated structural and electronic properties pertinent to the interaction of 1st and 2nd periodic-table elements, in addition to CO, with pristine and N/B defect mono-layer BN surfaces. Attachment of a CO molecule above a nitrogen vacant site dissociates the molecule in a reaction that occupies the vacant site with the carbon atom. Binding energies of the CO molecule noticeably varies with the adatom adsorbents for all considered BN structures. Analysis of electronic properties indicates that interaction of the adsorbate atoms with surface mainly involves occupation of adsorbate p electrons. The orbital centres for the adsorbates were calculated from a total energy width up to the Fermi level. Creation of the B and N surface defects and the subsequent atomic adsorption on the surface are all thermodynamically feasible processes. Findings here shall find direct applications in efforts that target the wide-scale utilization of BN-monolayers in optical and catalytic applications where the high surface area BN serves as supports and the decorated atoms represents the active sites in chemical sensing and reactions. We anticipate that an interesting extension of the current work is to compute other important properties' most notably IR spectra for the various configurations.

## Conflicts of interest

There are no conflicts to declare.

## Supplementary Material

RA-013-D3RA05108K-s001
