# Supplementary material for: Monatomic reactions with single vacancy monolayer h-BN: DFT studies
Source: RSC Adv. 2023 Oct 16;13(43):30346–57. doi: 10.1039/d3ra05108k (PMC10578242; doi:10.1039/d3ra05108k)
Supplement: RA-013-D3RA05108K-s001 [file RA-013-D3RA05108K-s001.pdf]

Electronic Supplementary Material (ESI) for RSC Advances.
This journal is © The Royal Society of Chemistry 2023

Supplementary Materials (SM) for

Monatomic reactions with single vacancy monolayer *h*-BN: DFT studies

Nicholas Mondinos[1], Mohammednoor Altarawneh[2*], Amun Amri[3], Willey Yun Hsien Liew[4], Gerrard Eddy Jai Poinern[5], Zhong-Tao Jiang[1**],

[1]Surface Analysis and Materials Engineering Research Group, College of Science, Health, Engineering and Education, Murdoch University, Murdoch, WA 6150, Australia
[2]Department of Chemical and Petroleum Engineering, United Arab Emirates University, 15551, United Arab Emirates
[3]Department of Chemical Engineering, Universitas Riau, Pekanbaru, Indonesia
[4]Faculty of Engineering, Universiti Malaysia Sabah, Jalan UMS, 88400, Kota Kinabalu, Sabah, Malaysia
[5]Murdoch Applied Innovation Nanotechnology Research Group, College of Science, Health, Engineering and Education, Murdoch University, Murdoch, WA 6150, Australia

Corresponding authors emails: mn.altarawneh@uaeu.ac.ae*;Z.Jiang@murdoch.edu.au**

Implementation of VASP code

The BN monolayer is modelled on a (8x4) supercell consisting of 128 atoms with cell dimensions; **x** = 20.4478 Å, **y** = 17.7084 Å, **z** = 22.1739 Å. The calculations for adsorption energies had a maximum of three different atomic species (B, N, and Atom). To minimize errors, calculations for isolated atom, of the surface only, and of the (adsorbate + surface) are done with an identical supercell, energy cut-off and with the following VASP parameters:

ENCUT =570 eV (ensures it is ~ 1.3x E_{max}), PREC= Accurate, LREAL =Auto, LMAXMIN= 2, ISYM = 0 (not use symmetry), ISMEAR = 0 (Gaussian smearing; partial occupancies for each orbital), IBRION = 2 (CGA; ionic relaxation), IVDW = 1 and VDW_S6 = 0.75 (vdW correction), ENAUG =1214 and ROPT = $2x10^{-4}$ (for each different type of atomic species). The maximum values of E_{max} and ENAUG are derived from the atomic potential functions (in this case, O and C respectively).

Spin polarised calculation results

After structural relaxation was achieved, calculations for magnetic moments and local spin polarized electronic density of states (SDOS) were performed with a 12×12×1 κ-point mesh of 75 κ-points. Integration of magnetic moment and SDOS were calculated in the PAW sphere.

Figures S1 to S10 are the SDOS plots from the spin polarised DOS calculations, indicating orbital contributions from B, N, and adatom A (H, Li, C, O, Al, Si, P, S), of the full valence and conduction bands region for V_N, V_B and all A-V_N and A-V_B surfaces. The figures also include a magnification plot of the VBM/E_F/CBM region indicating any contributions from s- and p- orbitals from B, N atoms

and s-, p- , p_x, p_y, and p_z orbitals from the adatom atom. Since the magnitude of DOS for the adatom, in the full SDOS diagrams, is not visible at the scale of the plot, the magnitude of the adatom DOS contribution is multiplied by a factor ranging from 10 to 50. This is indicated in the legend of the plot *e.g.* (×20) means the magnitude of the raw DOS data, for the atomic species, has been multiplied by 20.

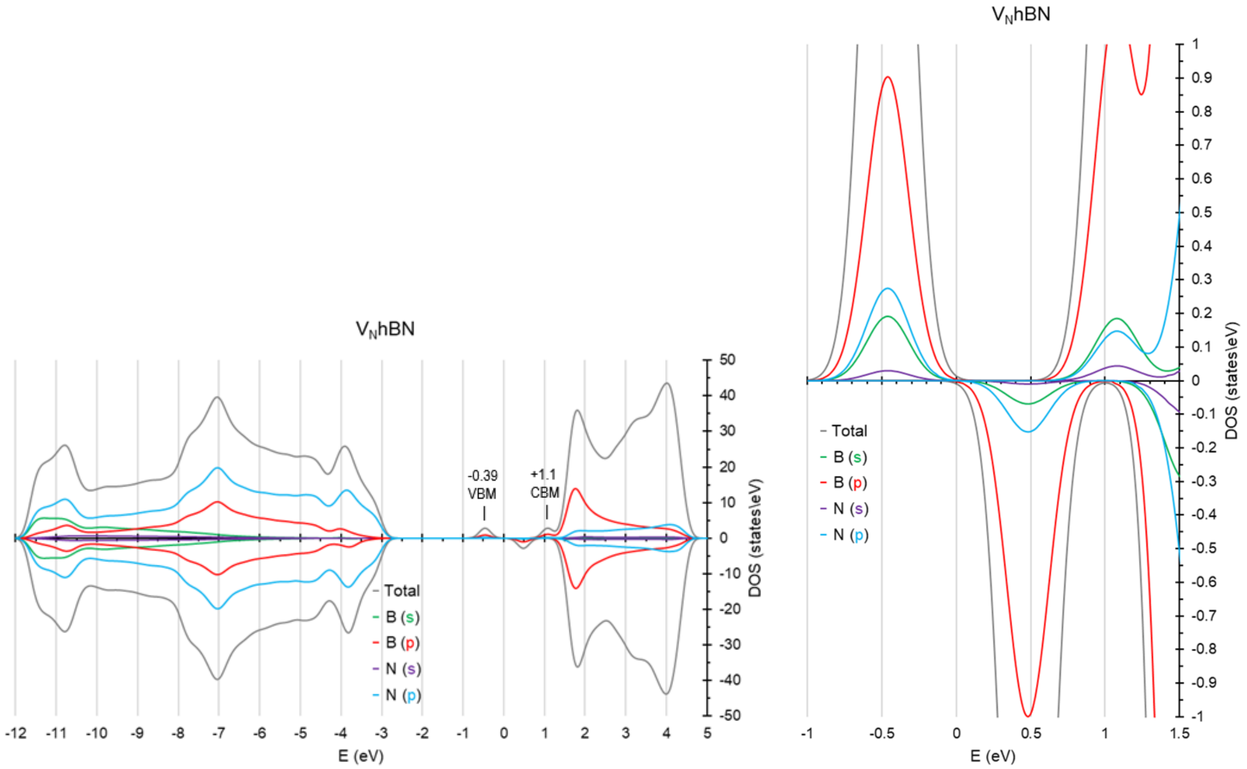

Figure S1 V_N - Full SDOS and magnification in the E_F region

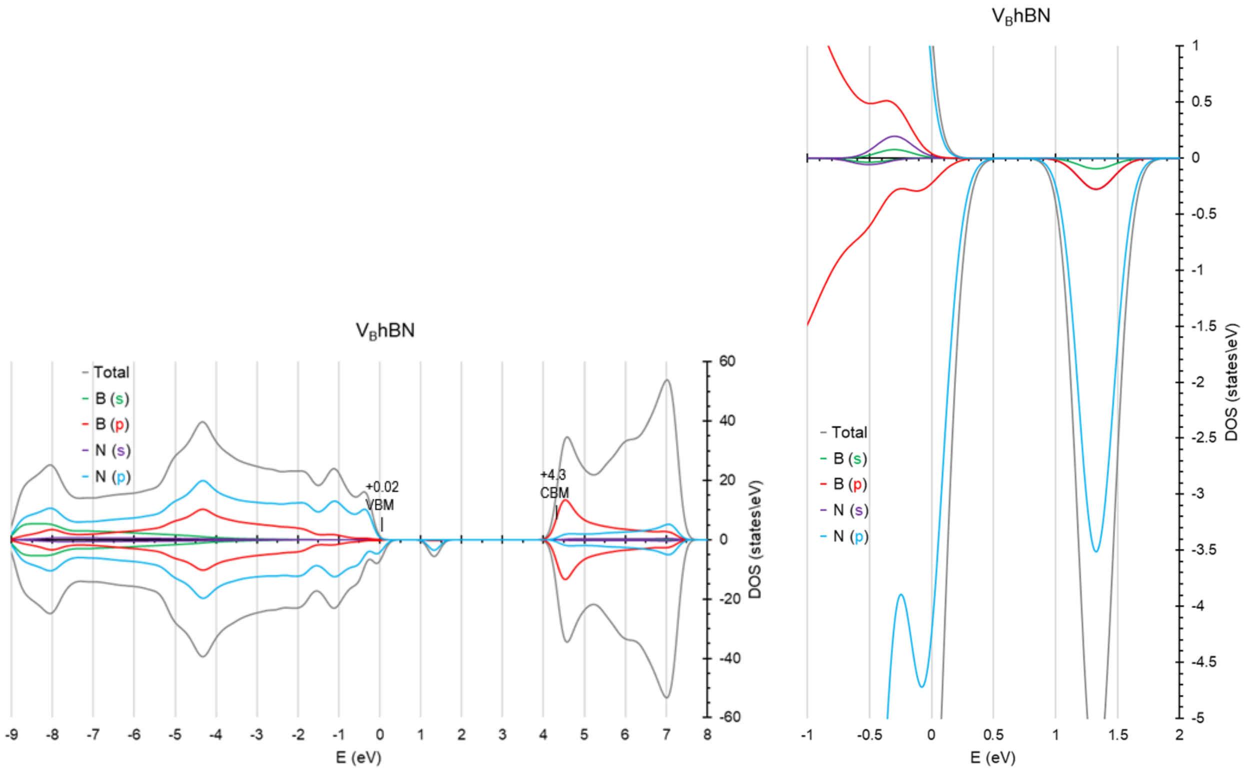

Figure S2 V_B - Full SDOS and magnification in the E_F region.

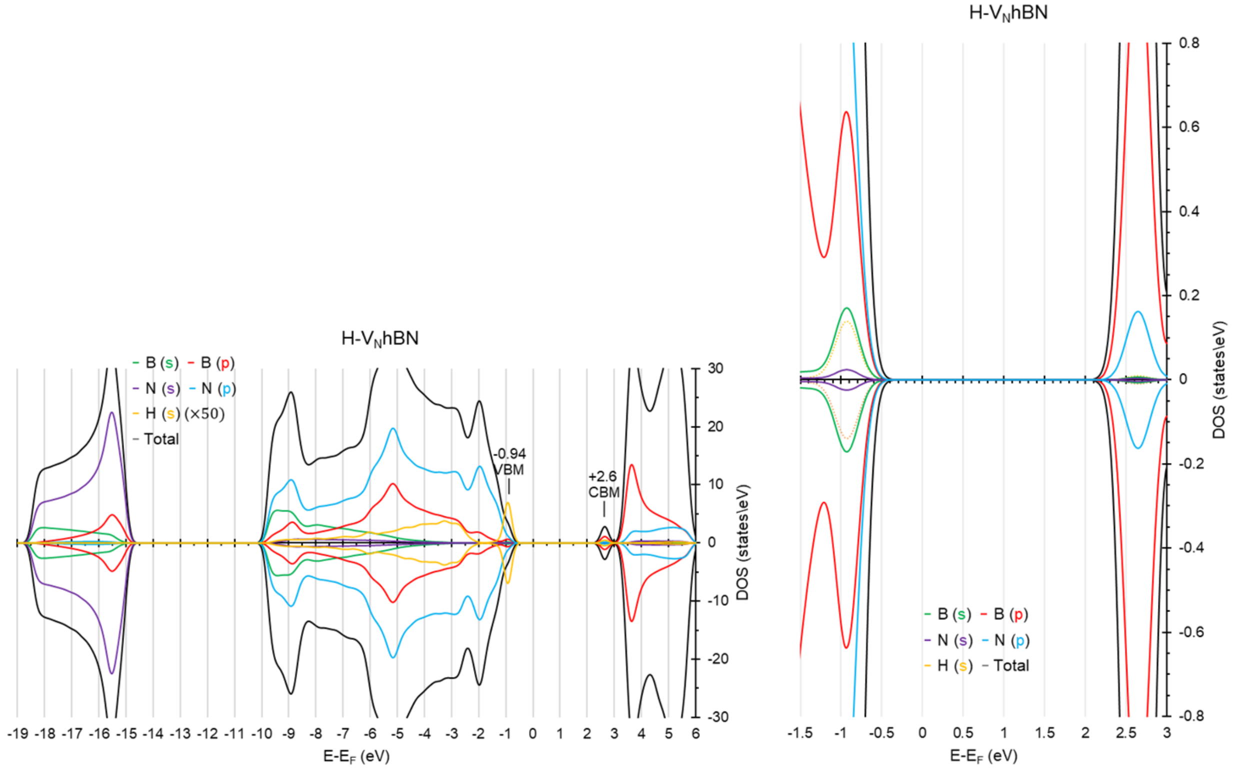

a)

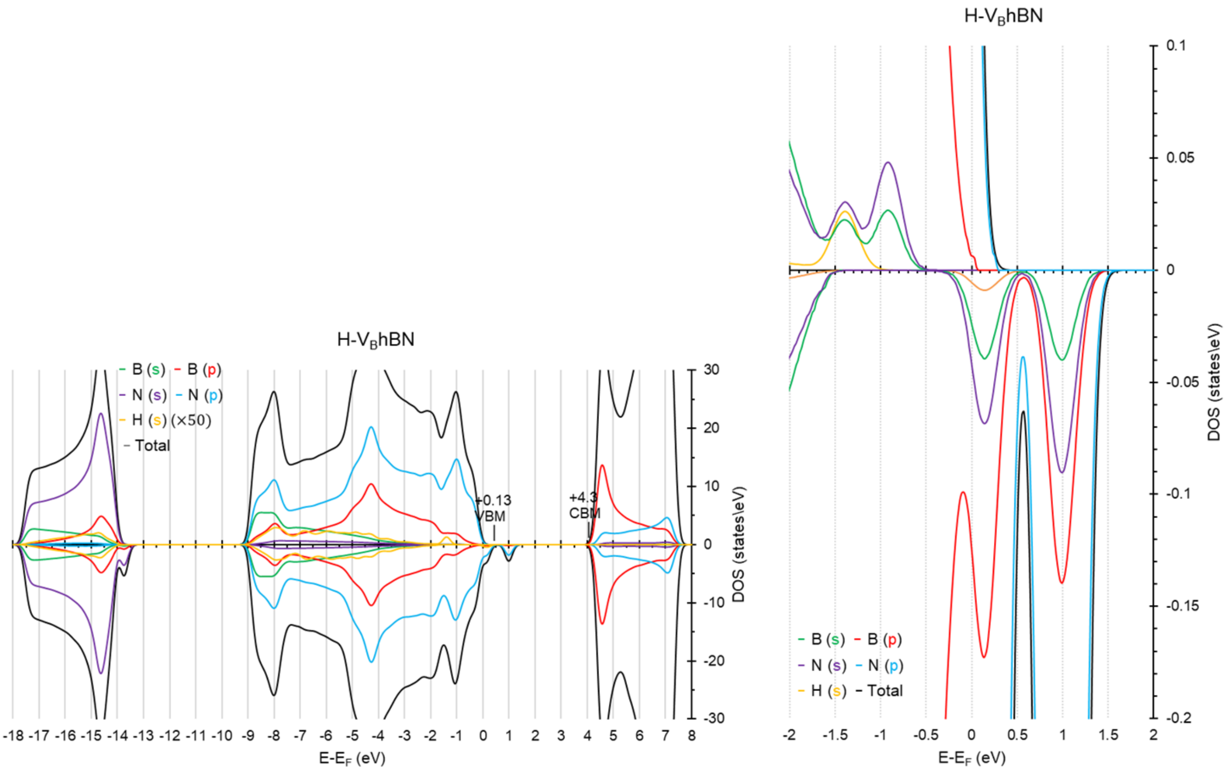

b)

Figure S3 (a) H-V$_N$ (b) H-V$_B$. Full SDOS and magnification in the E$_F$ region.

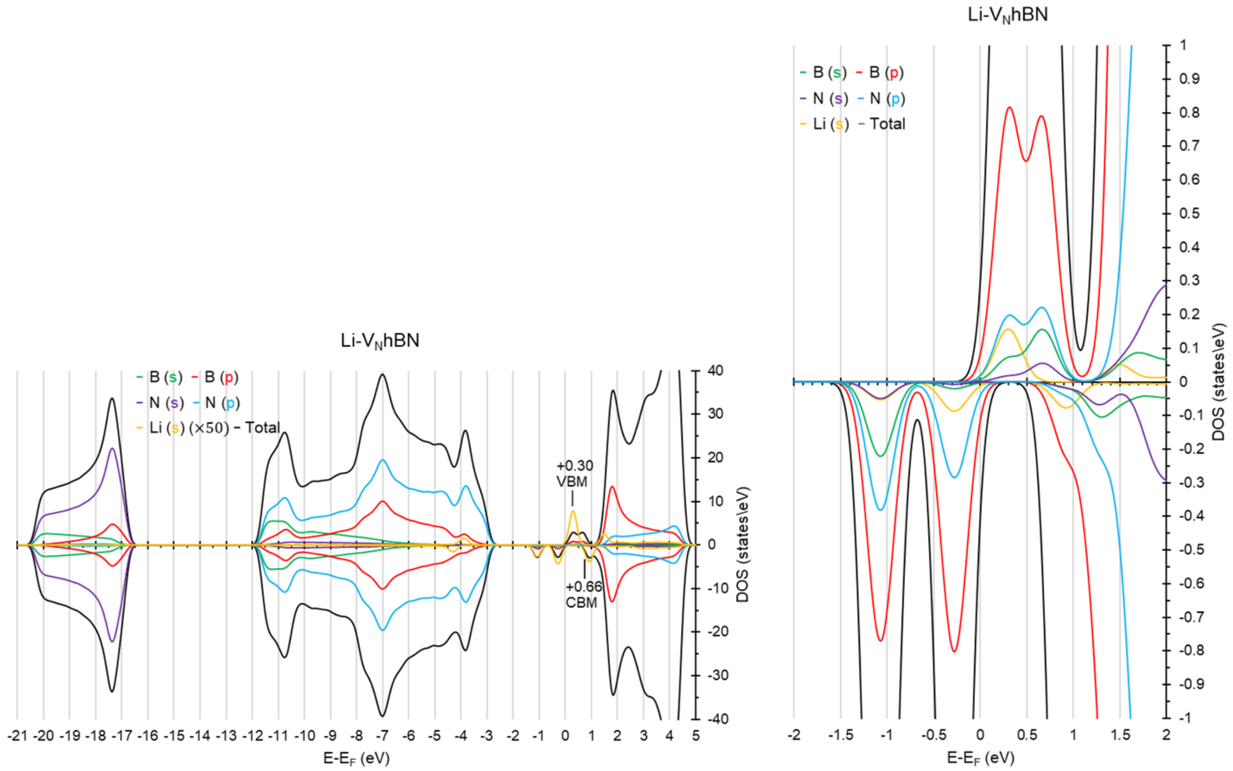

a)

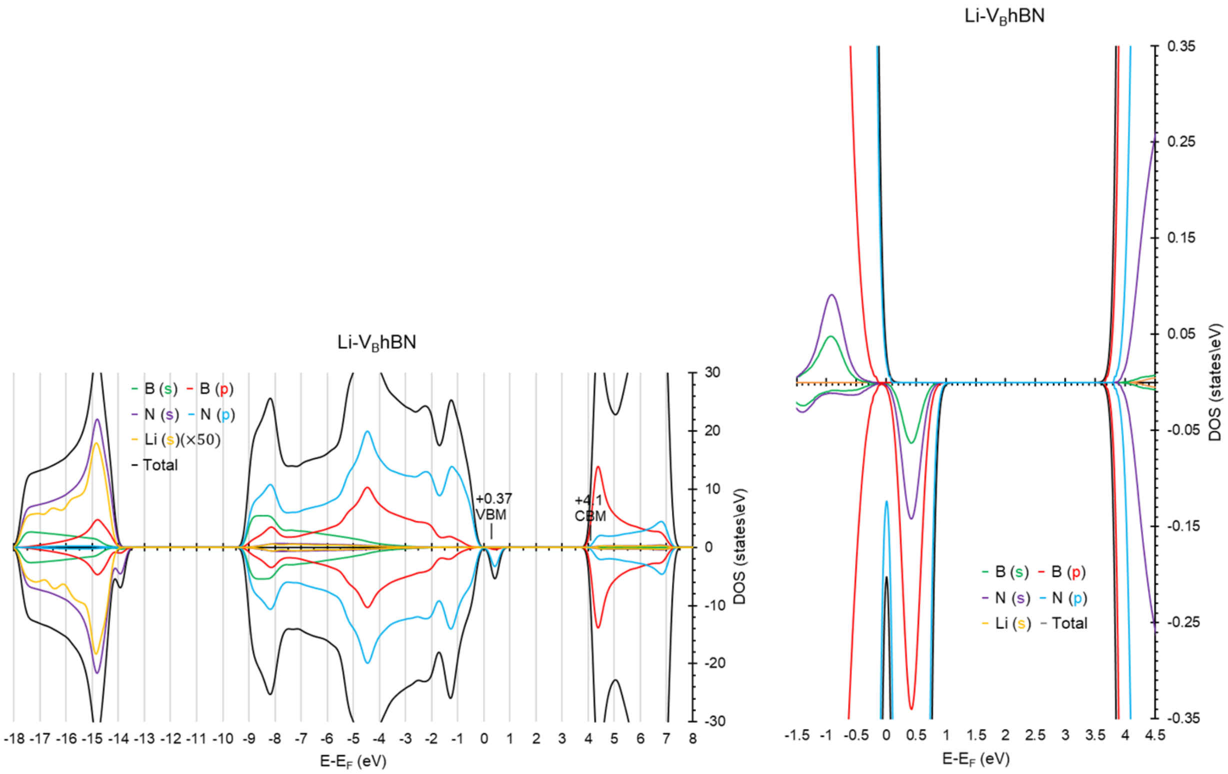

b)

Figure S4 (a) Li-V_N (b) Li-V_B. Full SDOS and magnification in the E_F region.

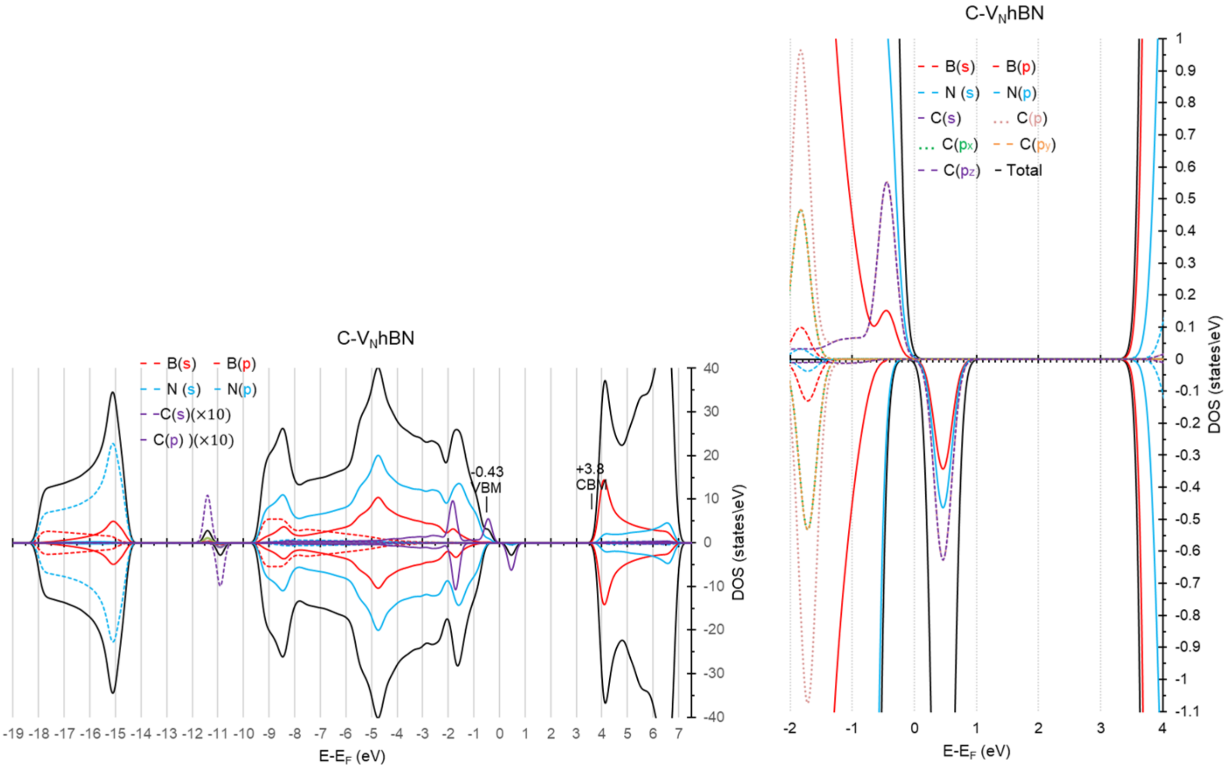

a)

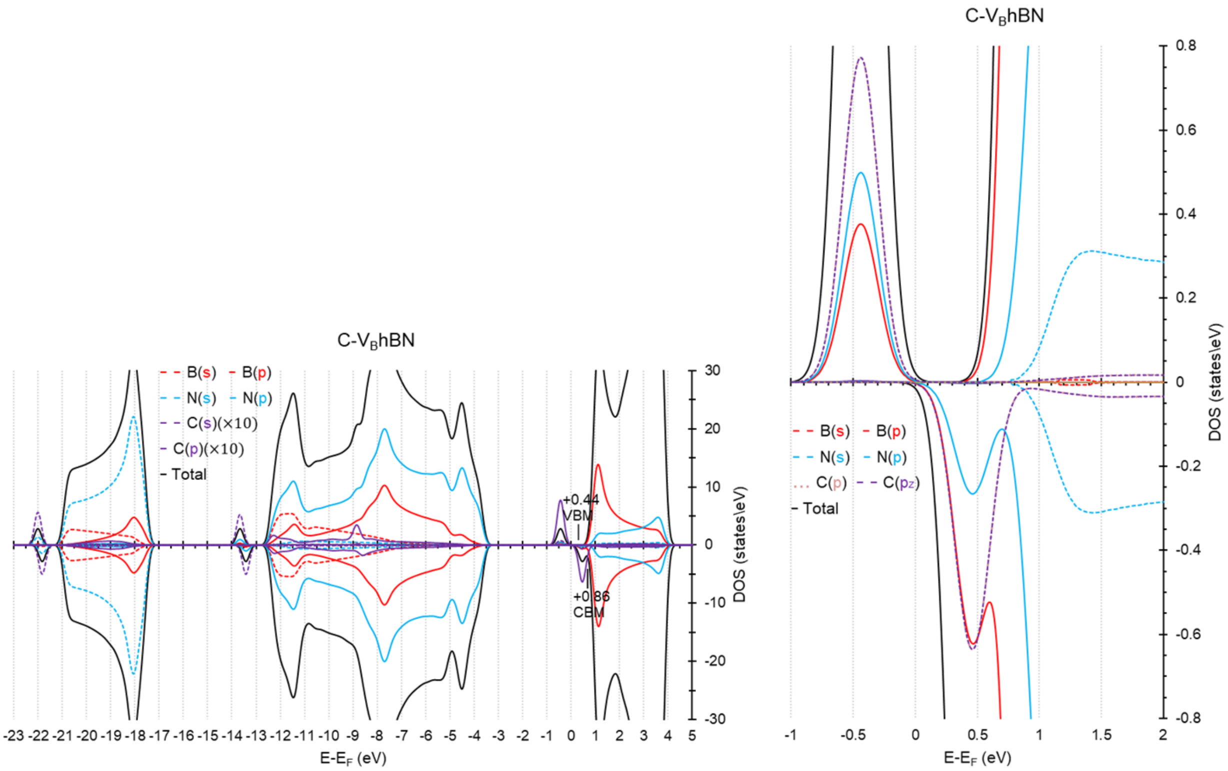

b)

Figure S5 (a) C-V_N (b) C-V_B. Full SDOS and magnification in the E_F region.

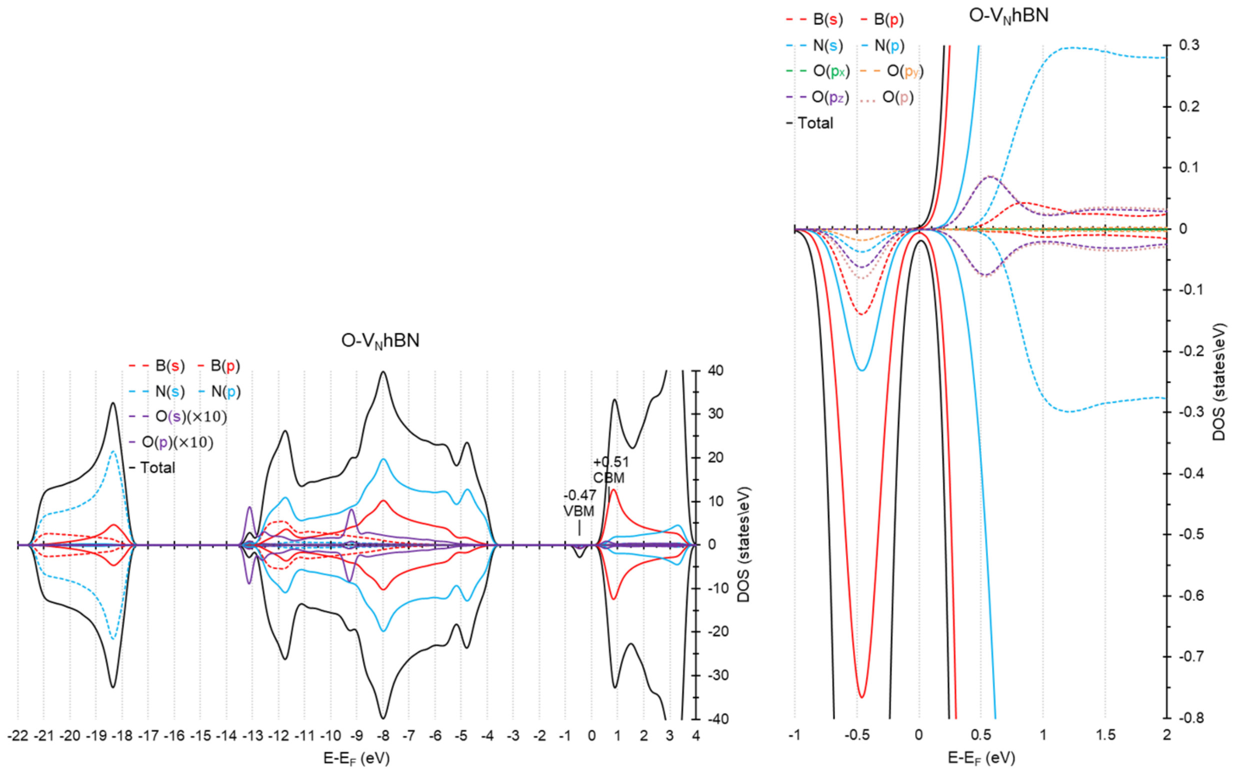

a)

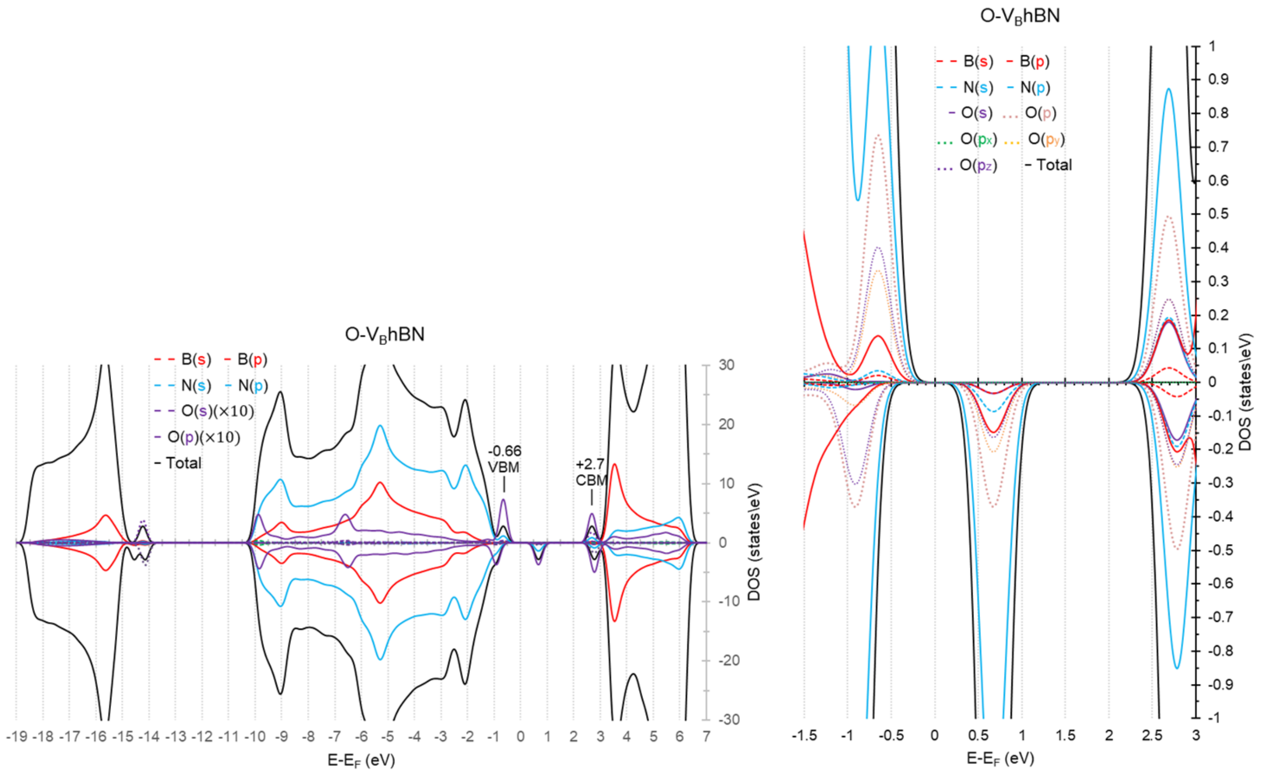

b)

Figure S6 (a) O-V_N (b) O-V_B. Full SDOS and magnification in the E_F.

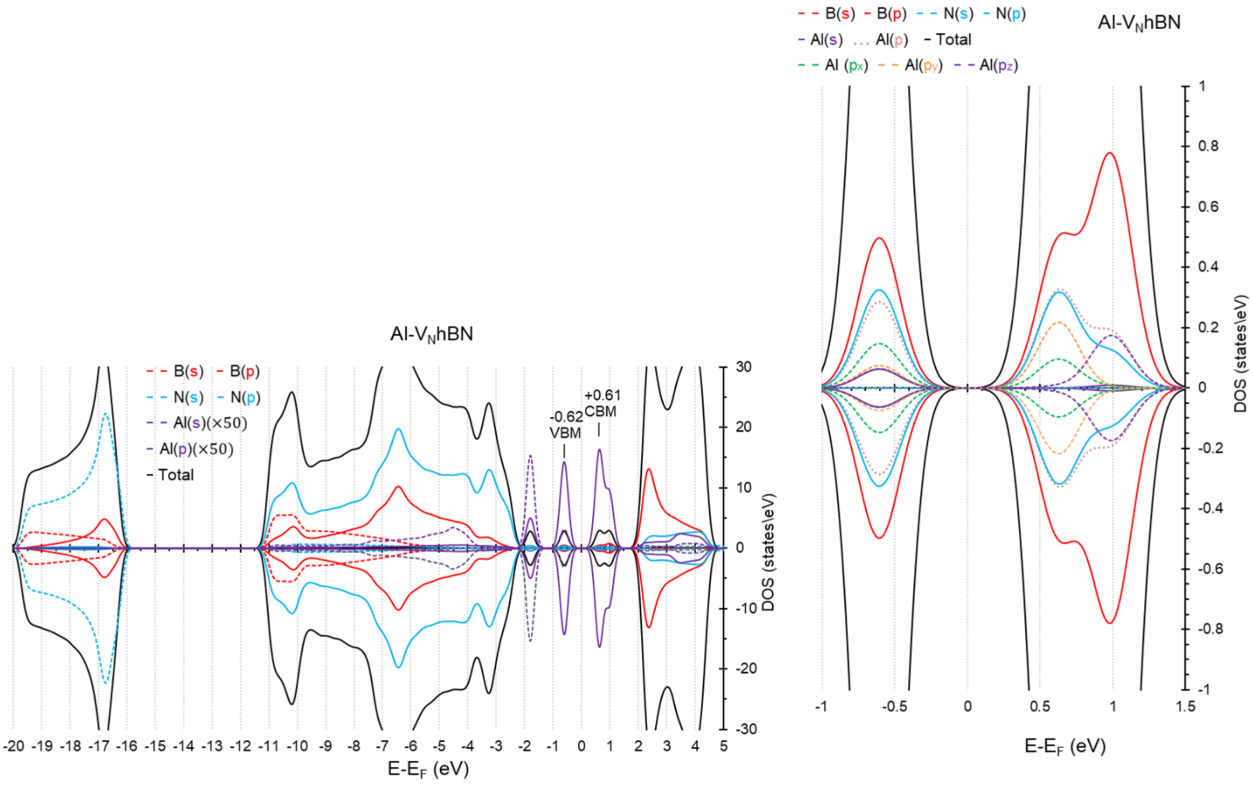

a)

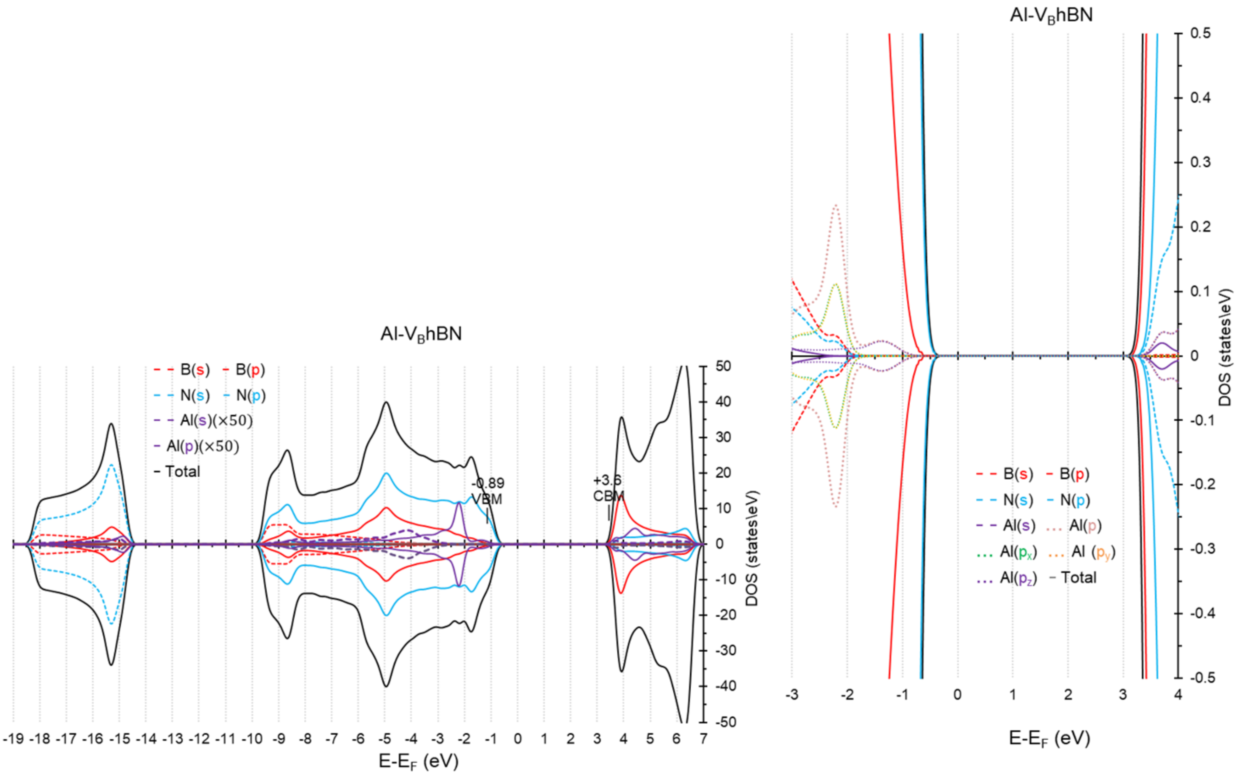

b)

Figure S7 (a) Al-V$_N$ (b) Al-V$_B$. Full SDOS and magnification in the E$_F$ region.

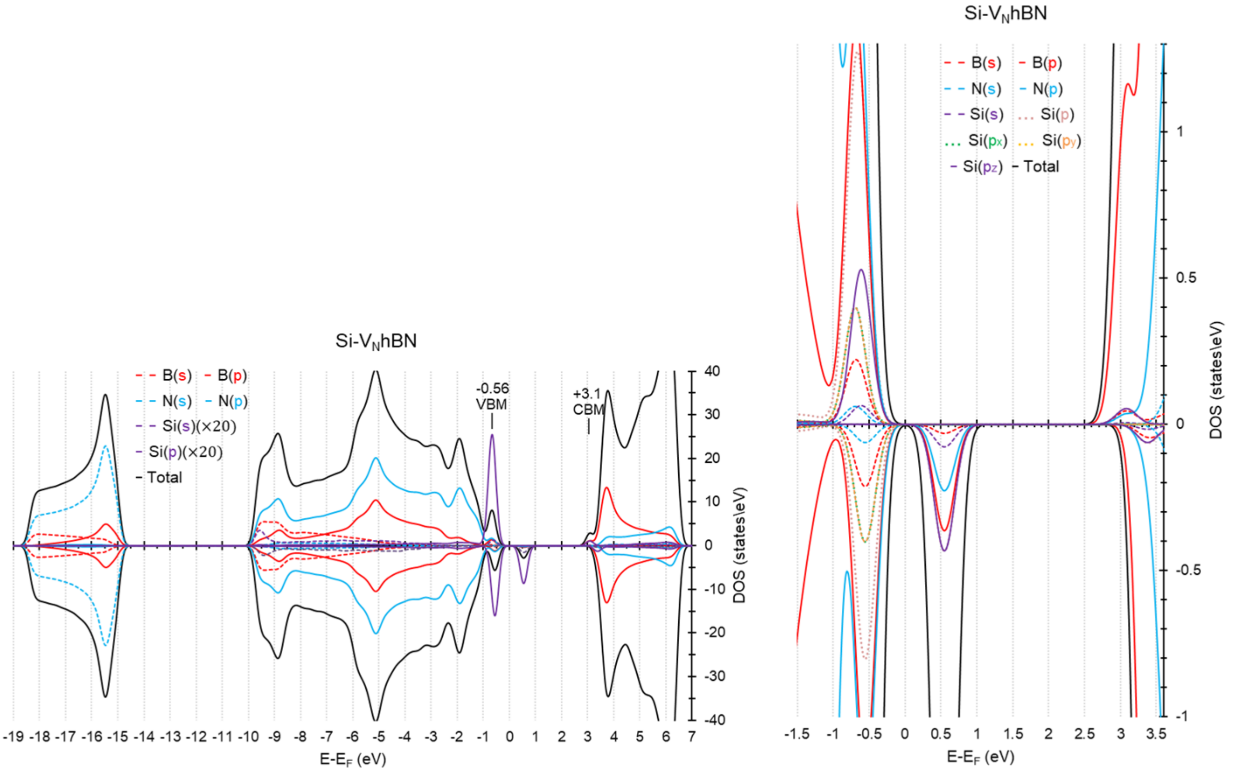

a)

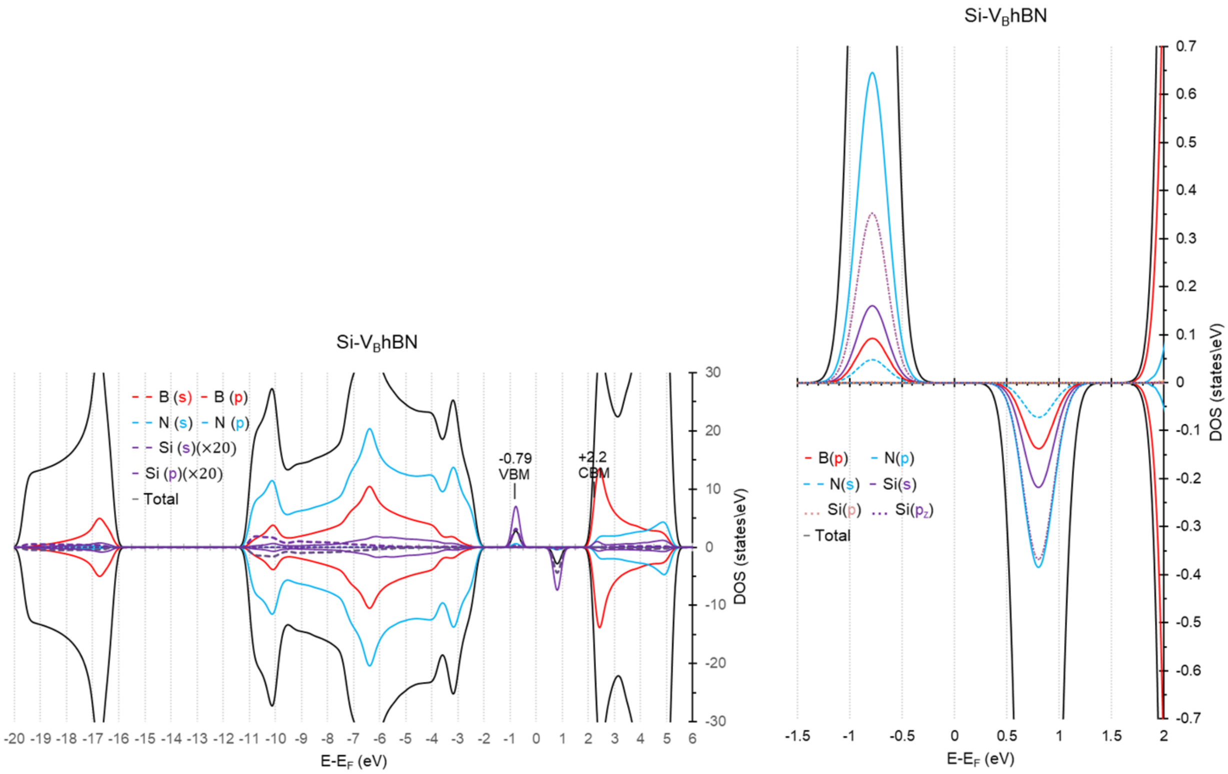

b)

Figure S8 (a) Si-V_N (b) Si-V_B. Full SDOS and magnification in the E_F.

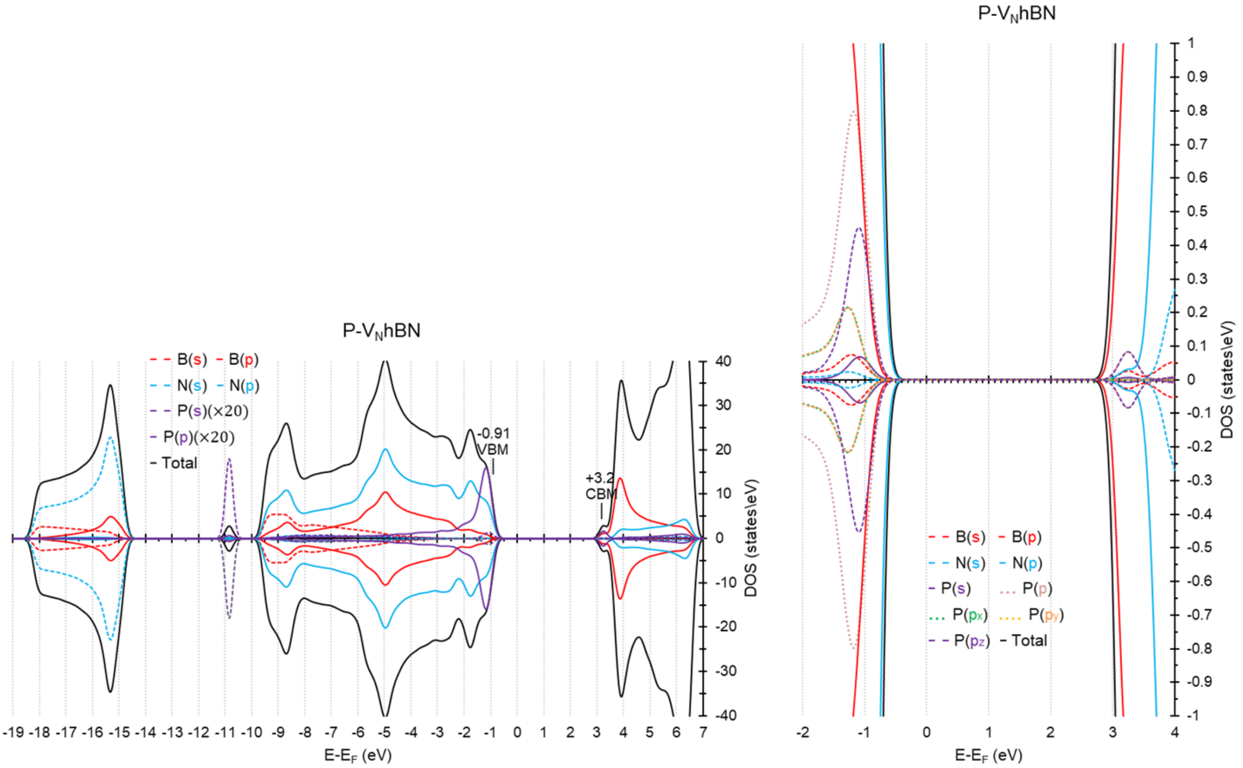

a)

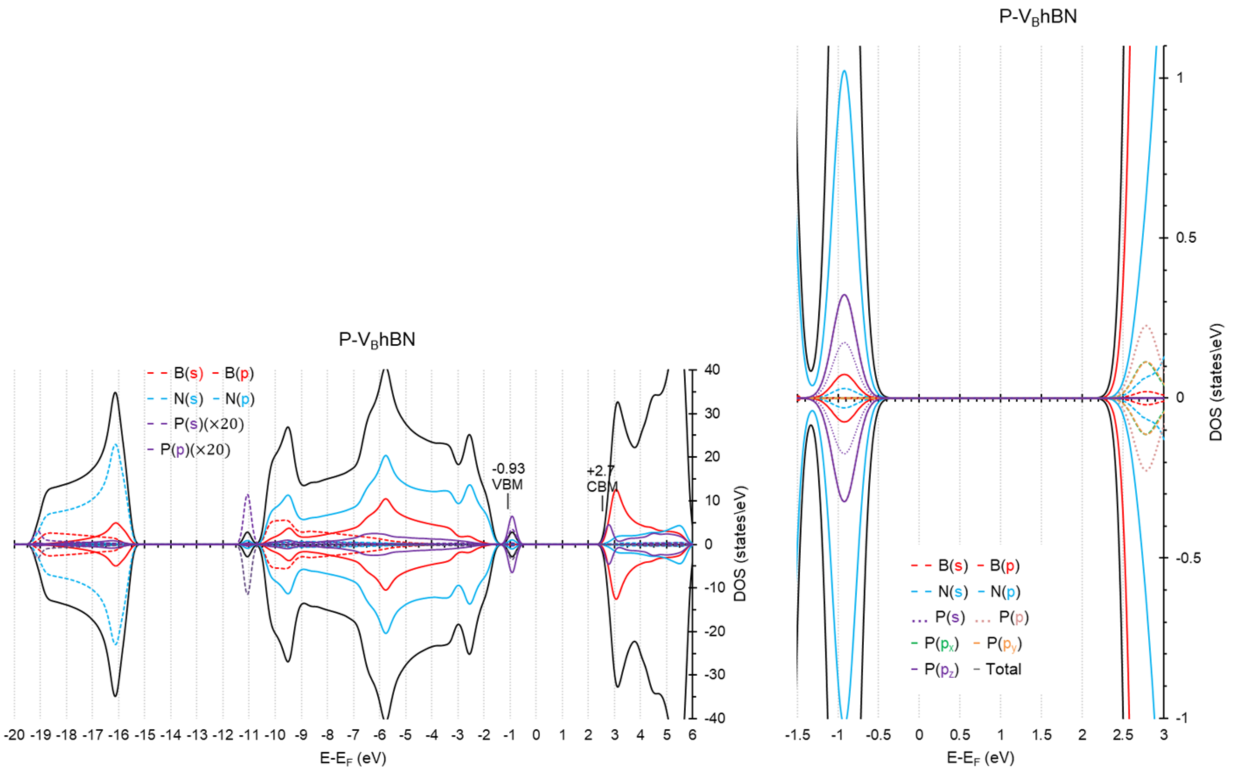

b)

Figure S9 (a) P-V$_N$ (b) P-V$_B$. Full SDOS and magnification in the E$_F$ region.

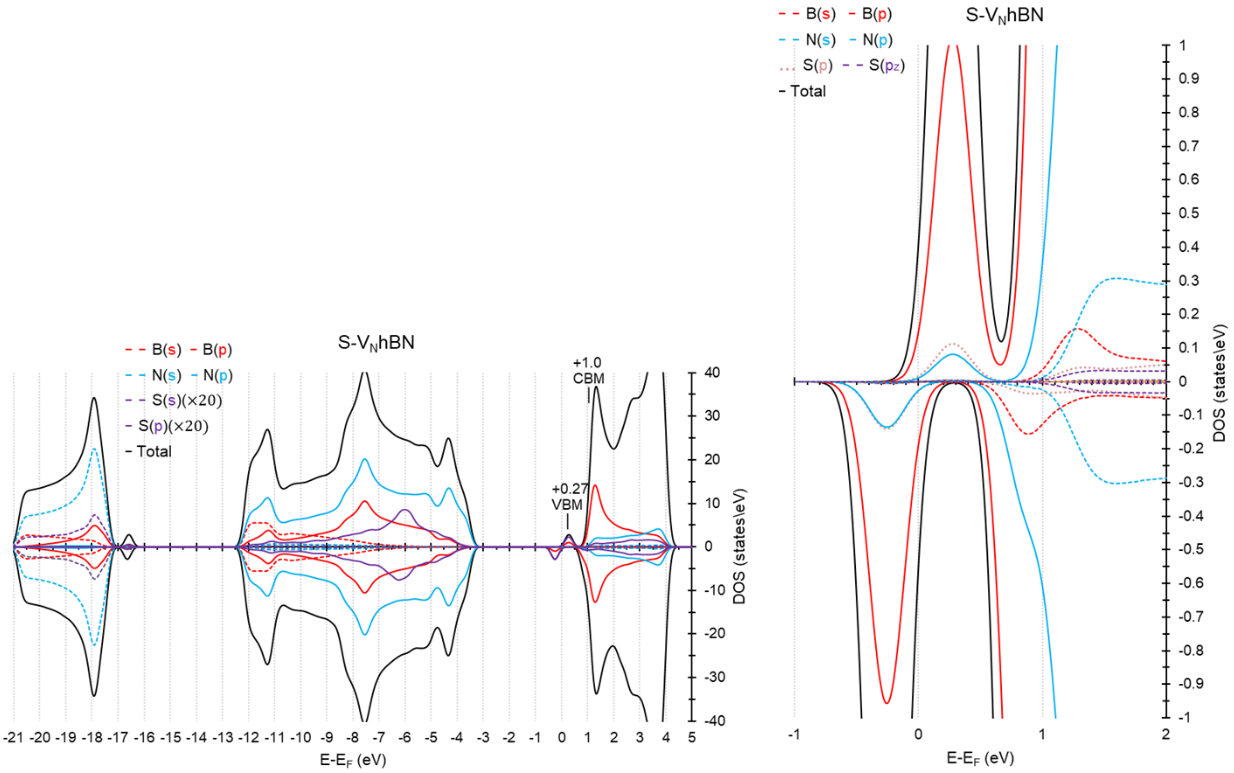

a)

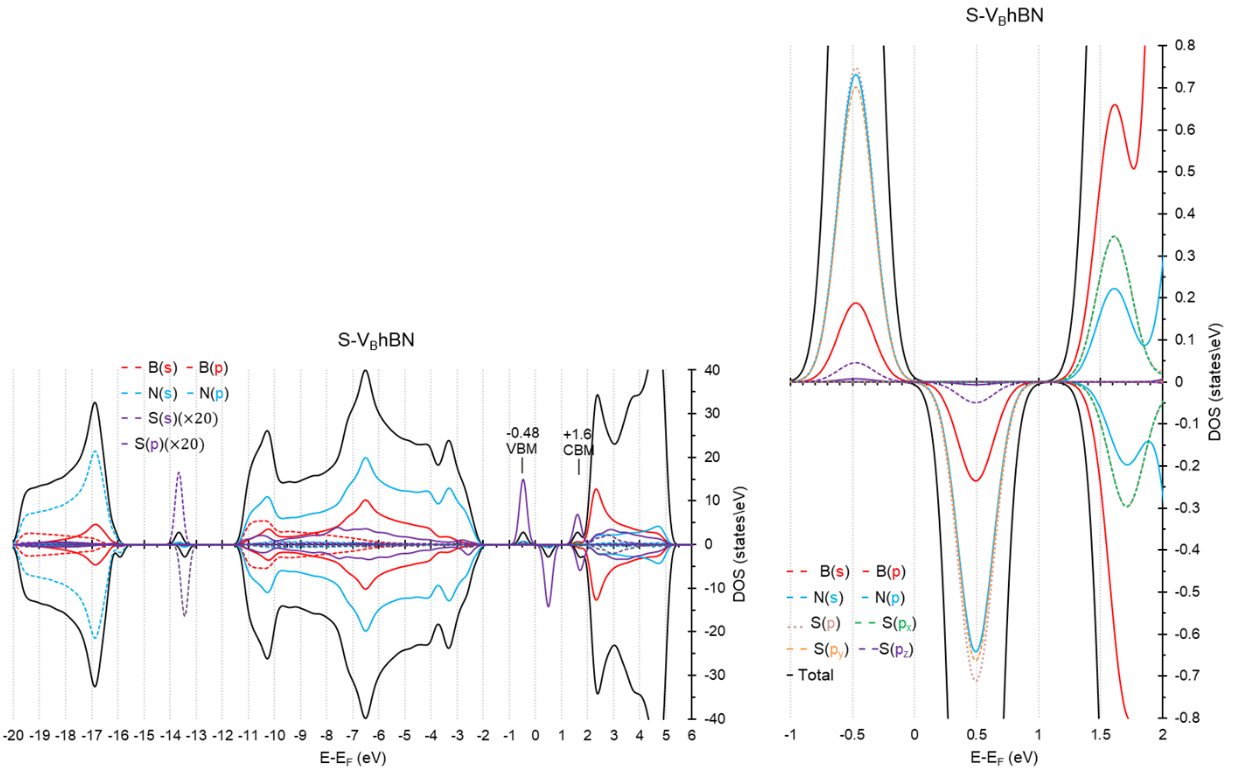

b)

Figure S10 (a) S-V_N (b) S-V_B. Full SDOS and magnification in the E_F region.

Average energy of p-orbital centers, ε_p

The ε_p energy values are with respect to E_F. The width gives the range of energy levels up to E_F. All units are in eV.

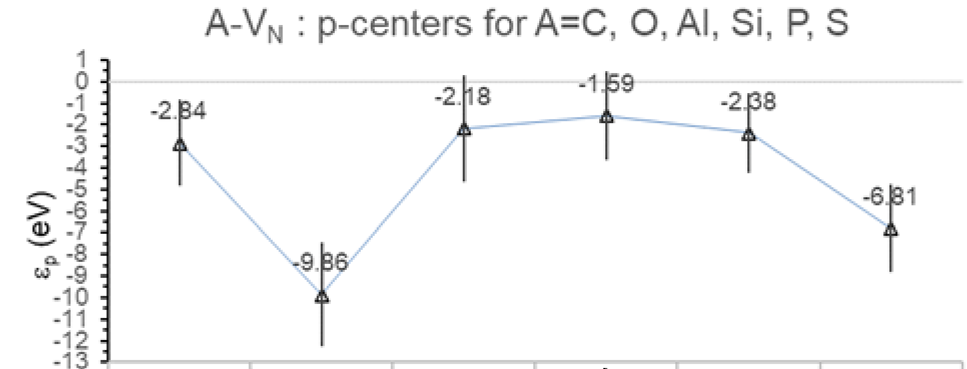

	C	O	Al	Si	P	S
STD	2.01	2.43	2.47	2.04	1.84	2.02
Width	-9.5	-13.5	-11.0	-10.0	-9.5	-11.5

Figure S11 A-V_N.

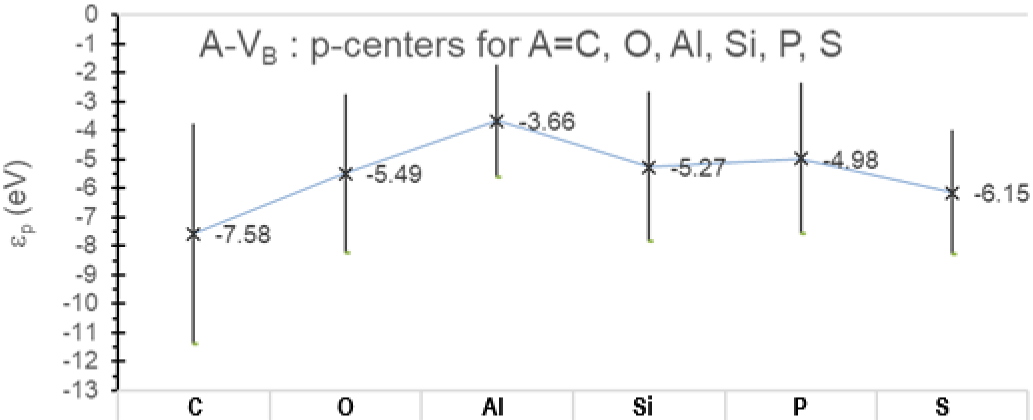

	C	O	Al	Si	P	S
STD	3.81	2.76	1.95	2.60	2.63	2.17
Width	-12.5	-10.0	-9.5	-11.0	-10.5	-11.0

Figure S12 A-V_B.
